# Characterization and Comparison of Microbiota in the Gastrointestinal Tracts of the Goat (*Capra hircus*) During Preweaning Development

**DOI:** 10.3389/fmicb.2019.02125

**Published:** 2019-09-13

**Authors:** Bibo Li, Ke Zhang, Chao Li, Xiaolong Wang, Yulin Chen, Yuxin Yang

**Affiliations:** College of Animal Science and Technology, Northwest A&F University, Xianyang, China

**Keywords:** microbiota, temporal-spatial specificity, gastrointestinal tract, preweaning, ruminant

## Abstract

Bacterial communities in gastrointestinal tracts (GIT) play an important role in animal health and performance. Despite its importance, little information is available on the establishment of microbial populations in the goat GIT or on changes occurring during early development. Therefore, this study investigated the bacterial community dynamics of the rumen, duodenum, jejunum, ileum, cecum, and colon in 15 goats at five developmental stages (0, 14, 28, 42, and 56 days old) by using 16S rDNA sequencing and quantitative real-time PCR technology. 940 genera were found to belong to 44 phyla distributed along the GIT. As a whole, the microbial richness and diversity showed a clear increasing trend as the kids aged and alpha diversity differed significantly among GIT compartments mainly occurring at middle day ages (14 and 28 days). Principal coordinate analysis indicated that the bacterial community displayed distinct temporal and spatial specificity along the GIT in preweaning goats. As kids aged, the phylum Firmicutes was replaced by Bacteroidetes in rumen, whereas Proteobacteria in the large intestine was displaced by Firmicutes. The phylum Proteobacteria was mainly present in the small intestine in older animals. In the rumen, taxa, such as *Bacillus* and *Lactococcus* decreased and *Prevotella, Treponema, Ruminococcus*, and unclassified Prevotellaceae increased with the age of kids. Furthermore, a lower proportion of taxa, such as *Lactobacillus* and *Bacteroides* was observed with higher abundances of both *Christensenellaceae_R_7* and *Ruminococcus* in duodenum and jejunum in older animals. In the large intestine, the microbiota displayed taxonomic dynamics with increases of *Ruminococcaceae UCG 005*, unclassified *Lachnospiraceae, Barnesiella*, and *Blautia* as kids aged. Predicted pathway analysis suggested that genes involved in amino acid metabolism, and translation were abundant in both rumen and duodenum, while genes involved in membrane transport and carbohydrate metabolism were enriched in the large intestine. These results indicate that both the microbial colonization process and potential function exert a temporal-spatial specificity throughout the GIT of goats. This study provides new insight into the temporal dynamics of GIT microbiota development during preweaning and will aid to develop strategies for improving animal health and downstream production.

## Introduction

The microbiomes of the gastrointestinal tract (GIT) of mammalian animals have been increasingly identified as a critical factor in animal health, development, and productivity (Holmes et al., 2012; Yeoman and White, 2014). The GIT microbiome is an intricate microecosystem, which mainly consists of bacteria, archaea, anaerobic fungi, and ciliate protozoa (Holmes et al., 2012; Falony et al., 2016; Mao et al., 2016). Mutualistic relationships between GIT microbiota and their mammalian hosts have been reported to play important roles in the host's nutritional metabolism, to increase the host's resistance to pathogenic bacteria, and to crucially contribute to the maturation of the immune system (Dolan and Chang, 2017; Espín, 2017; Parker et al., 2018). In ruminants, a typical example of these relationships in the rumen is the fibro lytic activity, which facilitates the conversion of plant fibers to soluble small compounds (acetate, propionate, and butyrate). Then, these compounds are absorbed and metabolized for the energy requirements of the animal and for other physiological purposes (Mackie, 2002; Jami et al., 2013; Mao et al., 2015). For animal husbandry, the GIT is of great significance due to the ability of its microbiomes to transform the heat energy stored in plant structural carbohydrates into animal products, such as meat, wool, and milk (Jami et al., 2013).

During the early development of ruminant life, the rumen of neonate kids is unfunctional due to the undeveloped volume and papilla; therefore, the sucked milk enters the abomasum due to the closure of the esophageal ditch by the reflex arc (Khan et al., 2011; Soest, 2018). Although, the milk can partially pass to the rumen and constitute the first substrate for microbial fermentation in the rumen (Dias et al., 2017; Yeoman et al., 2018). Previous studies have demonstrated that the establishment of the rumen microbiota was conductive to the maturity of structural and physiological function of the rumen due to their fermentative products, which are pivotal for the rumen wall villi (Jami et al., 2013). Concomitant with the solid-feed intake and increase of microbial activity, the rumen then becomes functional and improves its absorptive and metabolic capacity by increasing its surface area and volume (Rey et al., 2012; Steele et al., 2016; Meale et al., 2017). Gradual conversion of the ingested milk to a solid feed, a qualitative change occurs that transforms the kid from pre-ruminant to ruminant. Simultaneously, the main digestive site shifts from the intestine to the rumen as the metabolite changes from glucose to volatile fatty acid (VFA). In addition, with the emergence of the ruminant behavior, other digestive compartments, such as the small intestine also develop in response to changes of enzymatic activity from lactase to maltase (Khan et al., 2016; Dias et al., 2018).

Coupled with the development of physiological structure and function in GIT, the bacterial community abundance and diversity also show a sharp change as kids develop. Moreover, it has been reported that the dynamic of GIT microorganisms prior to weaning exerts a lasting impact on both adult ruminant health and animal products (Jewell et al., 2015; Dill-McFarland et al., 2017). The majority of researchers focused on the bacterial community in the rumen and the feces of young ruminants due to the conventional interest in the rumen as the main functional organ and the convenience of fecal sampling (Lopes et al., 2015; Wang et al., 2016c; Shen et al., 2017; Xu et al., 2017). Hence, for quite a long time, these sites have always been treated as representative of the GIT microbiota. However, a number of studies reported that the rumen or fecal microbiota cannot represent the other GIT compartments (de Oliveira et al., 2013; Zhao et al., 2015; Donaldson et al., 2016). Consequently, the bacterial community in various GIT segments is still poorly characterized. A recent study explored the composition and potential function of digesta- and mucosa-associated along the GIT in adult dairy cattle (Mao et al., 2015) and another two studies investigated the bacterial microbiota dynamics across the GIT in preweaning dairy calves (Dias et al., 2018; Yeoman et al., 2018).

The ruminal microbiota after weaning and the bacterial community of compound stomachs (reticulum, omasum, and abomasum) in pre-weaned goats have been studied before (Han et al., 2015; Lei et al., 2018). Nevertheless, the early colonization process of the bacterial community in integrated GIT requires further investigation. To fill this gap, this study characterized the dynamics of bacterial community across the GIT (rumen, duodenum, jejunum, ileum, cecum, and colon) of goat kids during preweaning development (0, 14, 28, 42, and 56 days old). This work increases the understanding of the spatial-temporal changes in the microbiota of kids during early development and provides a frame for the design of strategies for preferable intervention of the GIT microbiota toward improving health or products.

## Materials and Methods

### Ethics Statement

This study was carried out in accordance with the regulations of Instructive Notions with Respect to Caring for Experimental Animals, Ministry of Science and Technology of China. The protocol was approved by the Experimental Animal Management Committee of the Northwest A&F University (No. 2014ZX08008002).

### Animals and Sample Collection

This experiment was conducted in the Original Breeding Farm of the Shaanbei White Cashmere Goat experimental station of the Northwest A&F University. In this experiment, 15 female goats with similar age and weight were used. Estrus synchronization technology was adopted to ensure all kids were birthed at the same time. After birth, the kids were selected and randomly divided into five age groups (0, 14, 28, 42, and 56 days, *n* = 3 for each group) according to the sampling time point. For the 0 day-age group, newborn kids were separated from the ewes and euthanized immediately before they were suckled. In other groups, kids were housed together with their mothers in the same pen where they were solely fed with colostrum (0–3 days) or raw milk until 25 days of age. The kids were allowed access to complete formula granulated feed (Table S1) from 25 days in addition to breast milk. All kids were fed two equal portions of the diet at 8:00 and 18:00 and drank water *ad libitum* throughout the whole experiment. By the respective deadline, the kids of each age group were sacrificed without prior feeding. Kids were euthanized via injection of thiopental (0.125 mg/kg of body weight) and potassium chloride (5–10 mL). After execution, the enterocoel was opened and the rumen, duodenum, jejunum, ileum, cecum, and colon were separated with suture line to avoid reflux of digesta among adjacent regions. Then, relevant digesta samples were collected and homogenized, separately. Finally, the homogenized digesta from each gastrointestinal tract segment were snap-frozen in liquid nitrogen and stored at −80°C for subsequent DNA analysis.

### DNA Abstract, PCR Amplification, 16S rRNA Sequencing, and Data Processing

All digesta samples were thawed at 4°C and kept on ice during the whole extraction process. About 1 g (wet weight) of homogenized digesta sample of each GIT segment was used for total genomic DNA extraction. The DNA was abstracted by a bead-beating method, followed by SDS/GITC and phenol–chloroform isoamyl extraction. The solution was precipitated with isopropanol and sodium acetate, and then, the pellets were suspended in 50 μL of Tris-EDTA buffer. The DNA concentration was quantified using a Nanodrop spectrophotometer and stored at −20°C until further processing.

The V3-V4 regions of the bacterial 16S rRNA genes were amplified in triplicate by PCR (95°C for 2 min, followed by 25 cycles at 95°C for 30 s, 55°C for 30 s, and 72°C for 30 s and a final extension at 72°C for 5 min). The universal primer used in this amplification protocol was: 338F (5′-ACTCCTACGGGAGGCAGCAG-3′) and 806R (5′-GGACTACHVGGGTWTCTAAT-3′), with a target product of 468 bp (Xu et al., 2016). The PCR reaction was conducted in a 20 μL mixture containing 4 μL of 5 × FastPfu Buffer, 2 μL of 2.5 mM dNTPs, 0.8 μL of each primer (5 μM), 0.4 μL of FastPfu Polymerase, and 10 ng of template DNA. PCR products were excised from a 2% agarose gel. With regard to purification, an AxyPrep DNA Gel Extraction Kit (Axygen Biosciences, Union City, CA, USA) was used. Purified amplicons were pooled in equimolar and paired-end sequenced (2 × 300 bp) on an Illumina MiSeq platform according to standard protocols (Caporaso et al., 2012).

Raw reads of different samples were demultiplexed and quality-filtered using default parameters in Quantitative Insights into Microbial Ecology (QIIME, version 1.9.1) (Caporaso et al., 2010), with the following criteria: (1) The 300-bp reads were truncated at any site, receiving an average quality score <20 over a 50-bp sliding window; truncated reads shorter than 50 bp were discarded. (2) Reads with 2 nucleotide mismatches in primer matching or that contained ambiguous characters were removed. (3) Sequences that overlapped at least 10 bp were assembled based on their overlap sequences. Reads that could not be assembled were abandoned. Then, valid and clean sequences were assigned to bacteria using the cluster command in Mothur 1.3. The operational taxonomic unit (OTU), which is a most commonly used microbial diversity unit, was clustered with a 97% similarity cutoff using UPARSE (version 7.1) (Edgar, 2013). Chimeric sequences were identified and removed using UCHIME version 4.2 (Edgar, 2010). Alpha and beta diversities were calculated for downstream analysis of OTUs. Good's coverage, Chao, Ace, Shannon, and Simpson indexes were used to estimate bacterial richness and community diversity. Principal coordinate analysis (PCoA) was applied to visualize the dissimilarity of microbial communities among different age groups. Analysis of similarity (ANOSIM) was conducted to assess significant differences among samples using Mothur version 1.3 (Schloss et al., 2009). The data of the rumen digesta sample was obtained from our team (Zhang et al., 2019, unpublished) and the accession number in NCBI was SRP119700.

### Prediction of Microbial Function

In this study, phylogenetic investigation of communities by reconstruction of unobserved states (PICRUSt) was used to predict the molecular function of each sample. PICRUSt provides a valuable approach to infer the metagenomic potential of microorganisms by using 16S rRNA gene sequences (Langille et al., 2013). The presumable genes and their functions are pre-calculated in the database of the Kyoto Encyclopedia of Genes and Genomes (KEGG). With PICRUSt, the nearest sequenced taxon index (NSTI) was calculated to measure dissimilarity between the predicted metagenome presented here and reference genomes. When this value is low, it seems that PICRUSt perform well in predicting the genomes of the microorganisms in an environmental sample. The differences of predicted bacterial function among GIT regions were compared by PCoA using the R (v.3.5.3) software package.

### Quantitative Real-Time PCR Analysis

The copy numbers of the 16S rRNA genes related to the total bacterial populations was enumerated via real-time PCR, which was performed in triplicate with the SYBR Premix Ex Taq II assay kit (TaKaRa Bio Inc., Shiga, Japan) using a CFX96 Real-Time PCR Detection System (Bio-Rad Laboratories, Hertfordshire, UK). The primers for total bacteria were as follows: bacF (5′-CCTACGGGAGGCAGCAG-3′) and bacR (5′-ATTACCGCGGCTGCTGG-3′) (Metzler-Zebeli et al., 2013). The 20-μLreaction mixture contained 10 μL of 2 × SYBR Premix Ex Taq II, 1 μL of each primer (10-mM working concentration), and 2 μL of 10 ng DNA templates. Amplification was conducted one holding cycle at 95°C for 10 min for initial denaturation, and then 34 cycles at 95°C for 10 s followed by annealing at 58°C for 15 s and extension at 72°C for 20 s. Briefly, a standard curve was generated using serial dilutions of standard plasmid DNA, containing the 16S rRNA gene sequence. The real-time PCR efficiency ranged from 90 to 110%; negative controls without DNA template were run with every assay to assess the overall specificity.

### Statistical Analysis

The differences in the alpha diversity (Chao and Shannon indexes), the variance of relative abundance of bacterial taxa, the total copy number of bacterial 16S rDNA gene, and the relative abundance values of KEGG pathways affected by ages or GIT regions were analyzed using SPSS (SPSS v.20, SPSS Inc., Chicago, IL, USA) via one-way ANOVA with Tukey's *post-hoc* comparison based on the assumption of the additivity of effects, the normality of distribution and the homogeneity of variances. Correlation between bacterial taxa and KEGG pathways was evaluated by Spearman's correlation test using the R “heatmap” package. Significance was declared at *P* < 0.05.

## Results

### Data Acquisition and Analysis

In the present study, a total of 90 samples from the digesta of rumen, duodenum, jejunum, ileum, cecum, and colon were collected from five different age groups: 0-day-old newborns, 14-, 28-, 42-, and 56-days-old suckling kids. 3,366,283 valid sequences were obtained after quality filtering with an average of (37,403 ± 4,090) reads per sample. To facilitate unified analysis, the reads of all samples were normalized according to the minimum sequence in one sample. Based on a specialized criterion (≥97% nucleotide sequence identity between reads), the whole number of OTUs reached 4,064. Individual-based rarefaction curves were generated for each sample to assess whether the OTU coverage was sufficient to accurately describe the bacterial composition of each region (Figure S1). In addition, the Good's coverage exceeded 0.99 (Table S2). These results showed that the conducted sampling effort was adequate for the samples of all animals. A total of 940 bacterial genera belonging to 44 phyla were identified in all samples.

### Temporal Changes of Bacterial Microbiome in Rumen, Small Intestine, and Large Intestine

**Figure 1:**
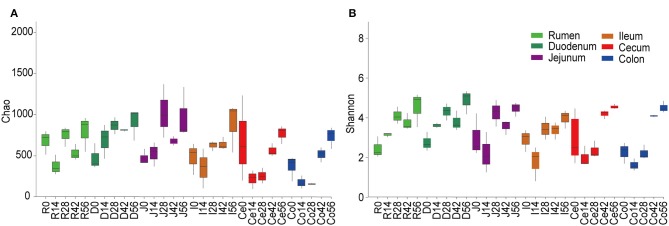
Alpha diversity of the bacterial community within each age group across the gastrointestinal tract. The richness and diversity were calculated via Chao **(A)** and Shannon **(B)** indexes, respectively. Boxplots indicate significant differences among ages within a given GIT region.

**Figure 2:**
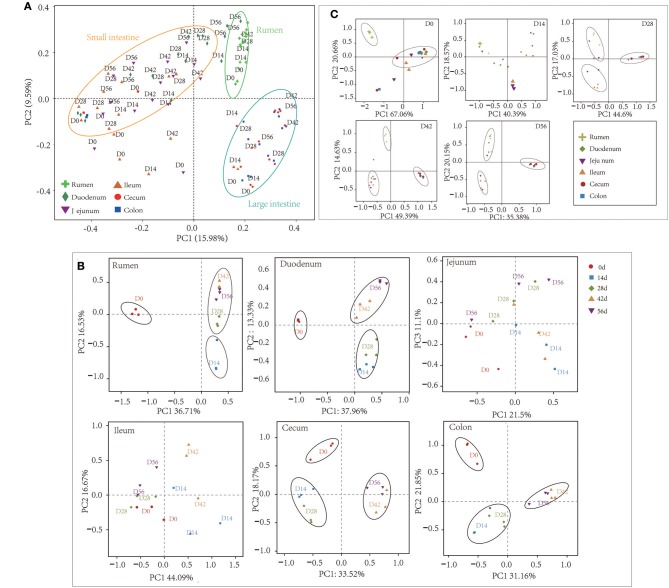
Principal coordinate analysis (PCoA) profile of microbial diversity using the Bray-Curtis dissimilarity metric. The percentage of variation explained by PC1 and PC2 are indicated on the axis. **(A)** PCoA of bacterial microbiota across all samples. **(B)** PCoA of bacterial microbiota according to age group in each GIT region. **(C)** PCoA of bacterial community according to gastrointestinal tract (GIT) regions for each age group.

At the 0.97 similarity level, the Chao and Shannon indices differed significantly among the age groups in the samples of all regions except for ileum and cecum (*P* < 0.001 to *P* < 0.05) (Figure 1, Table S3). The Chao value in the 14-days-old group was lower compared to other age groups in the samples of the rumen and colon, while in the duodenum and jejunum, it was lowest in the 0-day-old group (*P* < 0.05) (Figure 1A). With regard to the bacterial diversity, in rumen and duodenum samples, the Shannon index increased with increasing age. However, the Shannon index in samples taken from jejunum, ileum, cecum, and colon decreased in the 14-days-old group and increased significantly (*P* < 0.05) again in the three older age groups (Figure 1B). The bacterial composition similarity comparison of digesta samples by principal co-ordinates analysis (PCoA) of each age group using Bray-Curtis distance displayed that the samples clustered together based on their particular age group (Figure 2B). This was also evidenced via analysis of similarity (ANOSIM), which yielded significant and high R values between the age groups (Table S4). In the rumen, there were three sub-clusters, which consisted of the 0-day age group, the 14-days age group, and 28-, 42-, vs. 56-days age groups. This indicated that the bacterial communities in 28-, 42-, vs. 56-days age groups displayed a high degree of similarity. In duodenum, cecum, and colon, the samples clustered separately among each age group except for the 14- vs. 28-days age groups, and the 42- vs. 56-days age groups, suggesting that there were only higher similarities between both groups, respectively. However, the age showed no clear effect on bacterial composition in samples collected from jejunum and ileum.

**Figure 3:**
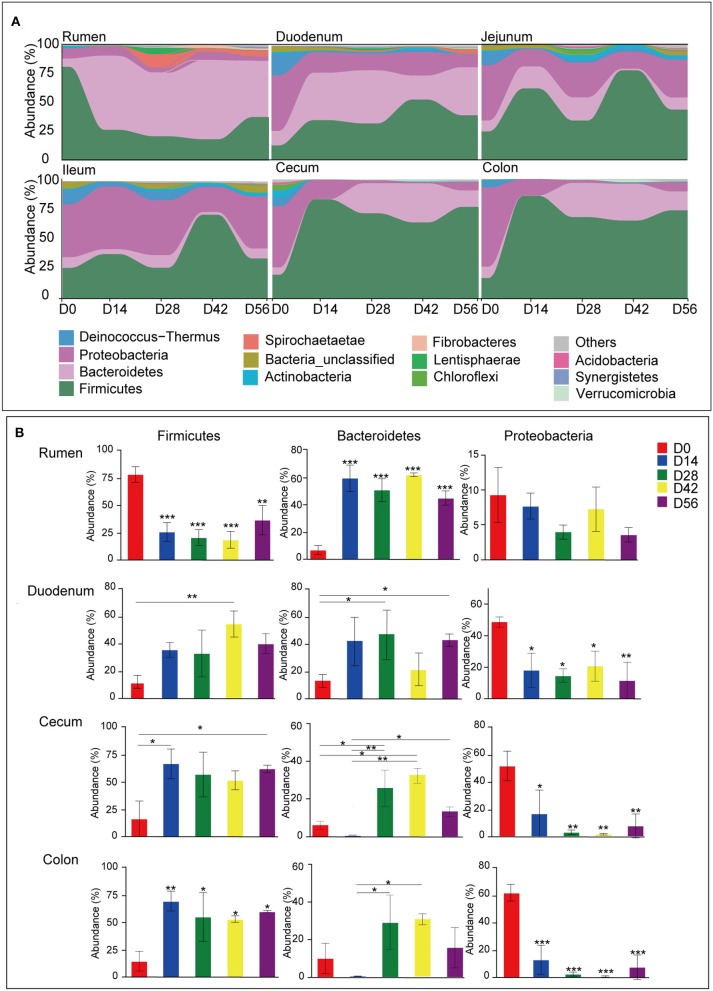
Microbiota composition at the phylum level. **(A)** Bacterial community composition changes as kids aged in each gastrointestinal tract (GIT) region. **(B)** Comparison of relative abundances of the three main bacterial phyla (Firmicutes, Bacteroidetes, and Proteobacteria) among age groups throughout the GIT region. Bars with a star symbol above their whiskers are significantly different between age groups in each GIT compartment using one-way ANOVA analysis; ^*^0.01 < *P* < 0.05; ^**^0.001 < *P* < 0.01; ^***^P < 0.001.

A total of 44 phyla were identified from digesta samples of all GIT in the five age groups (Figure S2). The majority of the sequences belong to Firmicutes (47.03%), Bacteroidetes (22.23%), and Proteobacteria (22.20%). Firmicutes dominated all microbiota along the length of the GIT apart from in the rumen and ileum, where Bacteroidetes or Proteobacteria were predominant, respectively (Figure 3A). In the rumen, the phyla Firmicutes found in samples taken from the 0-day group was extremely higher than the other four older age groups (*P* < 0.001), while there was an opposite trend in the cecum and colon (*P* < 0.05). The phylum Bacteroidetes was significantly lower in ruminal and duodenal samples taken from the 0-day group compared to other age groups (*P* < 0.05); however, Bacteroidetes became the most abundant phylum in samples of older animals. The proportion of phylum Bacteroidetes in cecal and colonic samples taken from the 28- and 42-days groups were significantly higher than that of the 0- and 14-days groups (*P* < 0.001). Compared to the 0-day group, Proteobacteria decreased radically in the 14-, 28-, 42-, and 56-days age groups (*P* < 0.001) (Figure 3B). The day-age had no significant effect on the phylum Firmicutes, Bacteroidetes, and Proteobacteria in samples taken from the jejunum and ileum. In addition, several minor phyla emerged in samples, such as Deinococcus-Thermus, Actinobacteria, Spirochaetae, Chloroflexi, Fibrobacteres, and Synergistetes, which could be found in all age groups along the GIT. The phylum Deinococcus-Thermus was more prominent in newborns in samples of all regions except for the rumen, where it was hardly found in each age group. The phyla Spirochaetae, Fibrobacteres, and Synergistetes were more abundant in older animals in samples collected from the rumen (Figure 3A).

**Figure 4:**
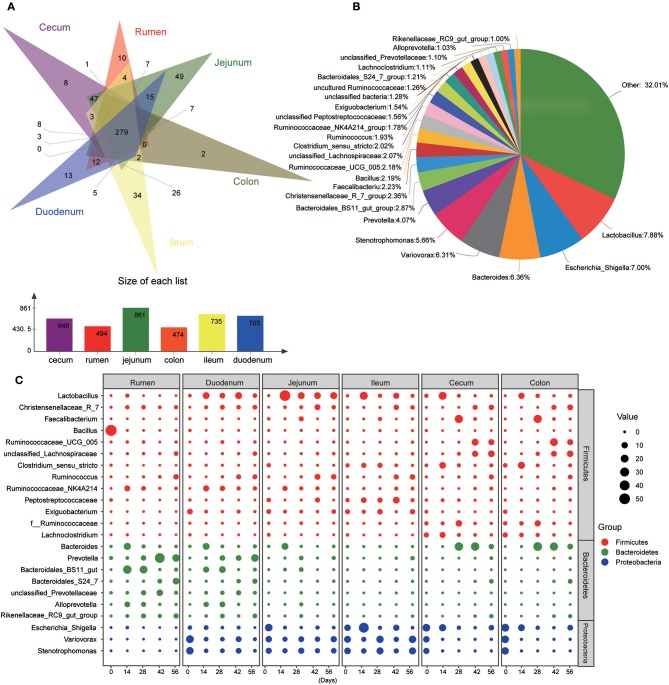
Main common genera across the gastrointestinal tract regions. **(A)** Venn diagram of bacterial genera shared between rumen, duodenum, jejunum, ileum, cecum, and colon. **(B)** Relative proportion of predominant common genera (those with an average proportion ≥1% based on all common genera were selected from 279 shared genera. **(C)** Changes of 23 common dominant genera as kids aged in each GIT region. The red, green, and blue dots represent genera that belong to the phyla Firmicutes, Bacteroidetes, and Proteobacteria, respectively. The size of the dot represents the relative abundance of microbiota.

At the genus level, 940 taxa were found in digesta samples taken from five age groups throughout the length of the GIT. The abundant genera (top 100) in each age group through the GIT sites (rumen, duodenum, jejunum, ileum, cecum, and colon) are shown in Figure S3. Interestingly, the genera *Bacillus, Terponema, Lactococcus*, and *Selenomonas* were mainly detected in ruminal samples but were extremely low in the gut. The genera *Hydrotalea* and *Sharpea* were mainly present in the small intestine while in the large intestine, the genera *Butyricicoccus, Subdoligranulum, Barnesiella*, and *Blautia* were highly percent. In addition, *Thermus* was mainly present in the small and large intestines. While performing Venn diagram analysis, 279 genera were found to be shared among the different GIT sites independent of the ages (Figure 4A). In these genera, 23 predominant genera (those with the average proportion ≥1% based on all common genera) were selected to elucidate the shaping process of the bacterial community with increasing age (Figures 4B,C). In the rumen, the genus *Bacillus* was extremely predominant in samples from the 0-day-old group compared to other four older age groups (*P* < 0.001), which reached up to 54.99%. In contrast, the genera *Prevotella* (*P* < 0.001), *Ruminococcus* (*P* < 0.05), and unclassified Prevotellaceae (*P* < 0.05) increased significantly with increasing age (*P* < 0.05). Several genera including *Ruminococcaceae_NK4A214_group* (*P* < 0.001), *Bacteroides* (*P* < 0.05), and *Bacteroidales_BS11_gut_group* (*P* < 0.01) were mainly present in samples from the 14- or 28-days groups. In the duodenal samples, the genera *Variovorax, Stenotrophomonas*, and *Exiguobacterium* in the 0-day-old group were significantly higher compared to all other groups (*P* < 0.001), while the genera *Lactobacillus, Christensenellaceae_R-7_group*, and *Ruminococcaceae_NK4A214_group* were mainly present in four older groups. With increasing age, the genera *Prevotella, Ruminococcus*, and *Bacteroidales_S24-7_group* increased significantly (*P* < 0.05). In addition, the genera *Bacteroides, Bacteroidales_BS11_gut_group_norank*, and *Alloprevotella* in the 14- or 28-days age group were notably higher than in other groups (*P* < 0.05). In the jejunum and ileum, the genus *Lactobacillus* increased sharply in the 14-days-old group, and then decreased in the other three older groups (*P* < 0.05). The genera *Christensenellaceae_R-7_group* and *Ruminococcus* increased significantly (*P* < 0.05) as kids aged. The genera *Bacteroides* and *Bacteroidales_BS11_gut_group_norank* were mainly found in the 14- or 28-days groups in the jejunum; while in the ileum, the genus *Clostridium_sensu_stricto* was mainly present in these two age groups. In the cecum and colon, the genera *Variovorax* and *Stenotrophomonas* were mainly present in the 0-day-old group compared to other older groups. The genera *Christensenellaceae_R-7_group, Ruminococcaceae_UCG-005*, and unclassified Lachnospiraceae increased prominently with increasing age (*P* < 0.05). The genera *Lactobacillus, Faecalibacterium*, and *Clostridium_sensu_stricto* were highest in 14- or 28-days groups, respectively (Figure 4C, Table S5). These results suggested that the microbial colonization process showed a clear temporal change and the bacterial genera along the GIT of goats can be divided into leading, transitional, and mature taxa.

### Bacterial Diversity and Composition Across Different Goat Gastrointestinal Tracts

For samples collected from the 14- and 28-days-old kids, the Chao and Shannon indices differed significantly among GIT (*P* < 0.05) (Figure S4, Table S6). In the 14-days age group, the Chao values in the small intestine (duodenum, jejunum, and ileum) were higher significantly than in the large intestine (cecum and colon), while the Shannon indexes in rumen and duodenum were significantly higher compared to in ileum and large intestine (*P* < 0.05). The Chao and Shannon indices in rumen and small intestine were significantly higher than in large intestine in the samples from 28-days age group (*P* < 0.05). However, the GIT had no significant influence on Chao and Shannon index in 0- and 56-days groups. In newborn kids, the Chao value in the large intestine was highest while the Shannon index was highest in rumen at 56 days. Furthermore, PCoA and ANOSIM were used to assess the bacterial composition similarity of samples among various GIT in each age groups (Figure 2C, Table S7). In the 0-day-old group, the bacterial composition in the rumen was spatially separated from that of the small intestine and large intestine, which corroborated the lower degree of similarity between rumen and intestine. There were three sub-clusters, consisting of samples in rumen and duodenum, jejunum and ileum, as well as cecum and colon in 28- and 56-days age groups. In the 42-days age group, bacterial communities of the rumen, small intestine and large intestine were spatially separated from each other. In summary, there was an unambiguous demarcation on the bacterial structure of samples from the rumen, small intestine, and large intestine (Figure 2A).

At the phylum level, the bacterial community composition also varied with different GIT sites in each age group (Figure S5A). In the 0-day-old group, the phylum Firmicutes was significantly higher in the rumen than in both the small and large intestine (*P* < 0.05). Firmicutes was most predominant in the large intestine while it was lowest in the rumen of the other four older groups. With regard to the phylum Bacteroidetes, in the 0-day-old group, no significant changes were found among different GIT sites. However, in the 14-days-old group, the phylum Bacteroidetes decreased significantly along the GIT (from rumen to colon) (*P* < 0.05). In 28-, 42-, and 56-days-old groups, the proportion of Bacteroidetes in the rumen was highest, while it was lowest in the jejunum and ileum (*P* < 0.05). The phylum Proteobacteria in the rumen was notably lower than in the small and large intestine (*P* < 0.05) in newborn animals. In the 28-, 42-, and 56-days groups, the phylum Proteobacteria in the small intestine was significantly higher than in the rumen and large intestine (*P* < 0.05). Moreover, the phylum Deinococcus-Thermus had a high proportion in small and large intestine in 0-day-old group compared to the rumen (Figure S5B).

At the genus level, in the 0-day group, the genus *Bacillus* in the rumen was extremely higher than in both the small large and large intestines (*P* < 0.01). However, the genera *Escherichia-Shigella, Variovorax*, and *Stenotrophomonas* were mainly found in the small and large intestines (*P* < 0.05). In the 14- or 28-days-old groups, the genera *Christensenellaceae_R-7_group* and *Ruminococcaceae_NK4A214_group* were more enriched in the rumen and duodenum (*P* < 0.05) while the *Clostridium_sensu_stricto* and *uncultured Ruminococcaceae* were mainly abundant in the large intestine. The genus *Faecalibacterium* was more enriched in large intestine in the 28-days group (*P* < 0.05). In the 42- and 56-days groups, the genera *Ruminococcaceae_UCG-005* and unclassified Lachnospiraceae were highest in the large intestine (*P* < 0.05). In addition, in the four older groups, the genera *Bacteroidales_BS11_gut_group_norank, unclassified Prevotellaceae*, and *Alloprevotella* were more enriched in rumen and duodenum, and the genera *Lactobacillus, Variovorax*, and *Stenotrophomonas* were mainly enriched in the small intestine (*P* < 0.05). It was noteworthy that the genus *Bacteroidales* was more enriched in the rumen and small intestine in the 14-days-old group whereas in the 28-, 42-, and 56-days old groups, it was highest in the large intestine (*P* < 0.05). Independent of the age group, the genus *Prevotella* decreased significantly whereas *Lachnoclostridium* increased throughout the GIT. The abundance of the genus *unclassified Peptostreptococcaceae* was highest in the ileum compared to other GIT regions (*P* < 0.05) (Figure 4C, Table S8). The obtained findings suggested that the microbial community displayed an evident spatial difference and the property varied with ages of kids.

### Predicted Function of Bacteria Based on 16S rRNA Gene Sequencing

**Figure 5:**
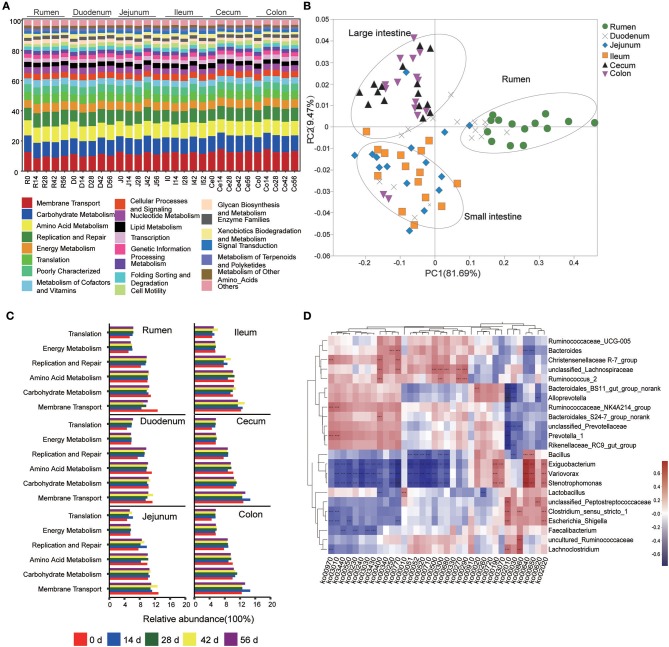
Metagenomic functional predictions. **(A)** Variations in KEGG metabolic pathways of microbiota at different ages throughout the gastrointestinal tract (GIT) region of kids. **(B)** PCoA profile of microbial functional KEGG pathways using Bray-Curtis dissimilarity metric according to the abundance of metabolic pathways. **(C)** Comparison of the predominant KEGG metabolic pathways in each age group. **(D)** Interaction heatmap between the predominant common bacterial genera and potential functional KEGG pathways (at the KEEG level 3); ^*^0.01 < *P* < 0.05; ^**^0.001 < *P* < 0.01; ^***^P < 0.001.

This study used PICRUSt as a predictive exploratory tool to investigate the molecular function of microbial communities. The average NSTI for 90 samples was 0.117 (Table S9). At the KEGG level 2, a total of 41 gene families were identified in all samples (Figure 5A). According to the relative abundances of the KEGG pathways of bacteria in samples, PCoA showed a clear distinction between the clustering of rumen, small intestine, and large intestine samples (Figure 5B). In these 41gene families, the majority of genes belonged to membrane transport (11.79% of total genes inferred by PICRUSt), carbohydrate metabolism (10.44%), amino acid metabolism (9.86%), replication and repair (8.76%), energy metabolism (5.65%), and translation (5.51%). Overall, for the six prominent gene families mentioned earlier, the proportion of the gene families associated with membrane transport increased enormously throughout the GIT (*P* < 0.001), while the relative abundance of genes involved in amino acid metabolism, replication and repair, and translation in the rumen were highest compared to those in small intestine and large intestine (*P* < 0.05) (Table 1). Moreover, the age played an important role on these gene families in specific sampling sites. In the rumen digesta sample, the abundance of genes related to membrane transport in 0-day-old group was signally higher than that in 14-, 28-, 42-, and 56-days-old groups (*P* < 0.05); however, the proportion of genes involved in replication and repair, as well as translation and energy metabolism in the 0-day-old group were lowest (*P* < 0.01). In the duodenal sample, the relative abundance of the genes related to the amino acid metabolism in the 0-day-old group was markedly higher than in the other four groups (*P* < 0.05), while the proportion of the genes linked to replication and repair and translation in the 0-day-old group were extremely lower compared to other groups (*P* < 0.001). The abundance of genes involved in translation in the 0-day-old group was also lowest compared to other groups in cecal and colonic digesta (Figure 5C, Table S10).

**Table 1:** Comparison of the six dominant KEGG gene pathways among different GIT regions.

**Category**	**GIT regions**						**SEM**	***P***
	**Rumen**	**Duodenum**	**Jejunum**	**Ileum**	**Cecum**	**Colon**		
Membrane transport	9.97^a^	10.53^a^	11.73^b^	12.19^bc^	13.22^d^	13.11^cd^	0.18	0.001
Carbohydrate metabolism	10.35	10.32	10.50	10.17	10.70	10.61	0.06	0.114
Amino acid metabolism	10.20^b^	10.17^b^	9.68^ab^	9.99^ab^	9.56^a^	9.54^a^	0.08	0.042
Replication and repair	9.42^b^	8.95^ab^	8.69^a^	8.37^a^	8.55^a^	8.57^a^	0.09	0.012
Energy metabolism	5.73	5.72	5.52	5.52	5.70	5.68	0.03	0.088
Translation	6.06^c^	5.68^bc^	5.46^ab^	5.17^a^	5.34^ab^	5.33^ab^	0.07	0.001

*Mean values with different superscripted lowercase letters within the same row differed significantly (P < 0.05). SEM, standard error of the mean*.

The KEGG level 3 showed that 251 KEGG ortholog (KO) pathways were present in all samples. Thirty-six prominent pathways (relative abundance ≥1%) were selected among these KO pathways, 63.9% of which belonged to carbohydrate metabolism (10 KOs), amino acid metabolism (8 KOs), and energy metabolism (5 KOs) (Table S11). To probe the link between microorganisms and functional genes, a genus-function interaction heatmap was constructed by calculating the Spearman's correlation coefficient (*R* > 0.5, *P* < 0.001) based on the abundance of 23 genera and 36 KOs. The genus *Lactobacillus* exhibited a positive correlation with the glycolysis/gluconeogenesis pathway (KO 00010) while it exhibited a negatively correlation with the glycine, serine, and threonine metabolisms (KO 00260). The genus *Bacillus* was positively involved in the propanoate metabolism (KO 00640) and butanoate metabolism (KO 00650) pathways but was negatively correlated with both galactose metabolism (KO 00052) and methane metabolism (KO 00680). The genera *Christensenellaceae R-7_group, unclassified Lachnospiraceae*, and *Ruminococcus* were positively involved in phenylalanine, tyrosine, and tryptophan biosynthesis (KO 00400). The pantothenate and CoA biosynthesis (KO 00770) was positively associated with the genera *Bacteroides*. Moreover, the genus *unclassified Lachnospiraceae* also positively participated in starch and sucrose metabolism (KO 00500), lysine biosynthesis (KO 00300), and valine, leucine, and isoleucine biosyntheses (KO 00290) while the genus *Ruminococcus* was positively correlated with cysteine and methionine metabolisms (KO 00270). The genera *Ruminococcaceae_NK4A214_group* and *Prevotella* were involved in aminoacyl-tRNA biosynthesis (KO 00970) and ribosome biosynthesis (KO 03010) but were negatively associated with ABC transporters (KO 00210). However, the genera *unclassified Peptostreptococcaceae, Clostridium_sensu_stricto, Lachnoclostridium*, and *Escherichia-Shigella* were positively correlated with the ABC transporter (KO 00210) pathway. In addition, the pyruvate metabolism (KO 00620) was positively correlated with the genus *unclassified Peptostreptococcaceae* but showed a negative correlation with the genus *Alloprevotella*. Intriguingly, the genera *Exiguobacterium, Variovorax*, and *Stenotrophomonas* were positively involved in oxidative phosphorylation (KO 00190), propanoate metabolism (KO 00640), butanoate metabolism (KO 00650), and the two-component system (KO 02020) while they were negatively correlated to a functional-gene cluster of 16 KOs. The latter mainly belongs to carbohydrate metabolism, amino acid metabolism, nucleotide metabolism, as well as replication, repair, and energy metabolisms (Figure 5D).

### Comparison of Total Bacterial Microbiota Among Each Goat GIT

Quantitative real-time PCR showed that the sampling sites and ages both significantly impacted the total bacterial populations by calculating the copy numbers of 16S rDNA gene (Table 2). The total bacterial numbers were highest in the rumen while they were lowest in the ileum (*P* < 0.001). The highest bacterial copy number was found in the 42-days-old group for all GIT sites (*P* < 0.001) except the rumen, where no significant difference was found. The lowest bacterial numbers were found in small intestinal samples in all age groups.

**Table 2:** Total bacterial numbers (log_10_ gene copies/g of digesta sample) attached to different GIT site from 0 to 56 d.

**GIT site**	**Age/d**					**G**	**SEM^1^**	***P*****-value^2^**		
	**0**	**14**	**28**	**42**	**56**			**S**	**A**	**G × A**
Rumen	–	10.22^C^	9.93^C^	10.62^C^	10.70^E^	10.37^D^	0.19	<0.001	<0.001	<0.001
Duodenum	6.61^aB^	7.20^bA^	8.08^cB^	7.90^cA^	8.61^dC^	7.68^B^				
Jejunum	6.63^aB^	6.79^aA^	6.83^aA^	8.79^cB^	7.62^bB^	7.33^B^				
Ileum	5.66^aAB^	6.40^abA^	6.67^bA^	8.70^cB^	6.49^abA^	6.78^A^				
Cecum	4.79^aA^	8.48^bB^	9.89^cdC^	10.39^dC^	9.14^bcD^	8.54^C^				
Colon	5.54^aAB^	9.63^bC^	10.43^bC^	10.38^bC^	9.39^bD^	9.07^C^				
A	5.85^a^	8.12^b^	8.64^c^	9.46^d^	8.66^c^					

*Mean values with different superscripted lowercase letters within the same row differ significantly (P < 0.05) while with different superscripted capital letters within the same column means differ significantly (P < 0.05)*.

*The blank represents that the DNA sample is not enough to perform the quantitative real-time PCR*.

1 *SEM, standard error of the difference of the means*.

2 *Probability of a significant effect due to GIT sites (G), day-old ages (A), and their interaction (G × A)*.

## Discussion

This study investigated the bacterial community dynamics along the GIT (rumen, duodenum, jejunum, ileum, cecum, and colon) during the preweaning development of goats. Overall, the bacterial community richness and diversity, measured by Chao values and Shannon index, increased as animals aged, which was consistent with previous reports (Rey et al., 2014; Dill-McFarland et al., 2017). This indicates that the microbial community in GIT may shift gradually toward a mature state as kids aged. However, in samples of rumen or large intestine, both the richness and diversity underwent a sharply change from 0 to 28-days-old, which may be mainly attributed to the animal's environment and the change of diet intake. Several studies report that the maternal vagina, skin and colostrum (or milk) can be as an early source of microbes to the neonatal GIT (Khodayar-Pardo et al., 2014; Ruiz et al., 2014; Gonzalez Moreno et al., 2016). In addition, the significant differences of the bacterial community richness and diversity among different GIT sections, which mainly occurred at middle day ages (at 14-, 28-, or 42-days-old), suggests that the microbiota in GIT presented a particular temporal-spatial specificity.

Generally, the results of PCoA profiling showed that the microbiota differed distinctly among rumen, small intestine, and large intestine (Figure 2A). This result is similar to a report on the bacterial microbiota distribution across the GIT in dairy cattle (Mao et al., 2015). Moreover, the PCoA and ANOSIM results in present study suggest that each age group has its characteristic microbiota along the GIT with the exception of the jejunum and ileum (Figure 2B and Table S3). Both the within-group similarity and the diversity index increased with age, implying that the bacterial microbiota undergoes a successive development of GIT colonization that coalesces toward a mature adult composition. It can be presumed that the bacterial community inhabiting the GIT of adult goats is more diverse and homogeneous, compared to that of neonatal kids, which seem to have more heterogeneity between different animals. Interestingly, similar observations have been reported for the bacterial microbiota of bovine rumen and human infants guts (Yatsunenko et al., 2012; Jami et al., 2013; Yeoman et al., 2018). In addition, these results indicate that the dynamics of the bacterial community exhibit high temporal heterogeneity along the different GIT sections (Figure 2B), and significant spatial difference was found with respect to the microbiota at each age (Figure 2C). This is consistent with a report on the bacterial community variation across the GIT in dairy calves (Dias et al., 2018). Generally, few genera were shared between the microbiota during the early development period and mature animals (Rey et al., 2012; Wang et al., 2016b; Lei et al., 2018). This partially arises from the undeveloped anatomical structure or incomplete physiological function of GIT (e.g., the rumen) during the first days of life due to the absence of the intake of solid feed or plant fibers. However, several of the genera, such as *Lactobacillus, Ruminococcus, Bacteroides*, and *Prevotella*, were mainly present in adults where they play a crucial role in fiber, starch, or protein degradation (Gänzle and Follador, 2012; Lengowski et al., 2016; Zeineldin et al., 2018; Wang et al., 2019). These also emerged in neonatal animals albeit at a minute percentage. This supports the model where the colonization of beneficial genera in the GIT occurs very early in life (even before the acquisition of colostrum) and is pivotal for their potential function in adult animals. These findings were confirmed by several previous studies (Jami et al., 2013; Dias et al., 2018; Lei et al., 2018), indicating that the establishment of functional genera begins when kids are exclusively fed colostrum.

The data presented in this study indicates that the bacterial community in different GIT regions is age specific. Age-related variations between the primary stages of development (0–14 days) and later stages (28–56 days) were most evident. This is likely due to the transition of diet, in which the solid concentrate and plant fiber increased gradually in addition to that of milk. In the rumen, the phylum Firmicutes, which was mainly composed of the genera *Bacillus* and *Lactococcus*, reached up to 54.99% and 13.16% of the total reads, was predominant in newborns, yet in older animals it was replaced by the phylum Bacteroidetes. This could be explained by previous studies, which reported that the delivery mode shapes the acquisition of the infant microbiome during the first days of life, i.e., the maternal vaginal microbiota are key for the establishment of bacterial community in vaginally born newborns (Yeoman et al., 2018). As the major components of the indigenous vaginal microbiota, the genera *Bacillus* and *Lactococcus* delivered to infants play a critical role in generating the organism's colonial resistance and in modulating the host's immunological effects (Shakhov et al., 2015). However, it has been pointed out that the colonization of maternal vaginal strains was transient and the genera capable of utilizing absorbable milk nutrients (e.g., *Bacteroides*) would replace these, which is consistent with the results of the present study (Dias et al., 2017; Ferretti et al., 2018). In several older age groups, distinct age effects were also observed in the rumen, as reflected by the fact that the genera *Ruminococcaceae_NK4A214_group, Bacteroides, Bacteroidales_BS11_gut_group*, and *Alloprevotella* whose dominance at 14 days was shifted to *Prevotella, Treponema, Ruminococcus*, and *unclassified Prevotellaceae* at 28 days, shortly after the availability of the solid concentrate feeds (Figure 4C, Figure S3, and Table S5). Likewise, an exploration of the dynamics of bovine rumen microbiota indicated that the genera (*Prevotella, Treponema*, and *Ruminococcus*), which mainly degrade plant fibers, increased, whereas single sugars, protein, and fat utilizers (*Bacteroides*) decreased as the diet shifted toward more fibers (Jami et al., 2013).

In addition, the ages of animals also significantly influenced the microbiota in the lower GIT (duodenum, jejunum, ileum, cecum, and colon) in kids. In the neonatal gut, the fast-growing facultative anaerobes, the majority of which belong to Proteobacteria, dominated all taxa due to the availability of high levels of oxygen (Guaraldi and Salvatori, 2012). In the present study, a similar observation was made where the phylum Proteobacteria (mainly including the genera *Escherichia-Shigella, Variovorax*, and *Stenotrophomonas*) dominated the gut at 0 day. Then, it was replaced by the phyla Firmicutes and Bacteroidetes, which then became the predominant taxa in older animals (Figures 3B, 4C). Interestingly, in the large intestine, the phylum Firmicutes acutely increased at the beginning of the 14 days, while the proportion of Bacteroidetes did not show a significant increase until 28 days. This may be attributed to the introduction of solid concentrate feed and plant fiber in addition to milk as goats aged. Moreover, the phylum Proteobacteria still remained at a relatively constant level in the small intestine as kids aged, which is likely because the members of this phylum can tolerate the adverse combined effects of the unique circumstances in the small intestine, such as the higher acidity, higher concentration of oxygen and antimicrobials (Donaldson et al., 2016; Kong et al., 2016; Espín, 2017). In contrast, an increase in saccharolytic and amylolytic genera was observed, which consisted of the genera *Christensenellaceae_R_7* (Morotomi et al., 2012) and *Ruminococcus* (Flint et al., 2012; Cann et al., 2016) throughout the whole gut, the genera *Prevotella* (Chiquette et al., 2008; Ramayo-Caldas et al., 2016) in the duodenum, and the genera *Ruminococcaceae_UCG_005, unclassified Lachnospiraceae, Barnesiella*, and *Blautia* in the cecum and colon (Dehority and Orpin, 1997; Chen et al., 2017; Mancabelli et al., 2017a,b), as kids aged and ingested more concentrate feed and fibers (Figure 4C, Figure S3). In preweaning goats, the rumen is either un- or under-developed and the colostrum or milk, which is the sole dietary source in kids prior to solid feed intake, often bypass the rumen and flow directly into the abomasum due to the closure of the esophageal groove (Dill-McFarland et al., 2017). During the preweaning stages, it is therefore essential to supply solid feed for kids to promote the development of GIT; hence, the colonization of the saccharolytic taxa in the lower gut is pivotal for efficient post-ruminal starch or fiber utilization. This is supported by recent studies that reported that the utilization of starch in the small intestine (62%) was largely attributed to microbial fermentation rather than to host enzymes, while this biological process of starch degradation occupied 37% in the large intestine (Gilbert et al., 2015; Liu et al., 2017). Accompanied by the increase of amylolytic taxa, the genera *Bacteroides, Lactobacillus, Butyricicoccus*, and *Faecalibacterium* (large intestine) decreased in older animals. Consistent with the results of the present study, these transient genera, which were associated with a primarily milk diet, also decreased in the GIT of calves since calves consumed more starter solid feed with age (Dill-McFarland et al., 2017; Dias et al., 2018). In summary, a reverse relationship between the afore-mentioned bacteria and the solid feed intake can be inferred. The genera *Lactobacillus*, as a representation of the phylum Firmicutes in the present study, usually utilize simple sugars (e.g., pentoses, hexoses, and lactose) or starch to produce lactic acid or acetic acid and ethanol (Whitman, 2009). As butyrate producers, the genera *Butyricicoccus* and *Faecalibacterium* can convert acetic acid to butyrate and release butyrate close to the epithelium, which promotes the proliferation and differentiation of the intestinal epithelium (Foditsch et al., 2014; Geirnaert et al., 2014). However, the fermenting ability of these bacteria is often limited by the availability of their preferred substrate, which will be out-competed by fiber-degraders with increasing plant fibers in the diet.

In addition to the microbial dynamics of aging kids, differences in bacterial taxa across the GIT were also observed. The findings of this study indicate that each GIT region inhibited its own species although common bacterial taxa were found along the GIT (Figures 3, 4). A similar observation has been reported previously (Wang et al., 2016a; Dias et al., 2018; He et al., 2018; Kim et al., 2019). In neonatal animals, the phylum Firmicutes was abundant in the rumen whereas the phylum Proteobacteria dominated all bacterial taxa in the gut. However, in older animals, the phylum Bacteroidetes became most dominant in the rumen and the phylum Firmicutes was predominant in the large intestine. Simultaneously, the abundance of the phylum Proteobacteria was highest in the small intestine (Figure S5). Corresponding to the variations of bacteria at the phylum level, common bacterial genera belonging to the above-mentioned phyla also varied significantly across different GIT (Table S5). Moreover, the obtained results suggest the existence of a distinct bacterial community in each compartment independent of the kids age, such as the genera *Lactococcus, Treponema*, and *Selenomonas* in the rumen, the genera *Hydrotalea* and *Sharpea* in the small intestine, and *Subdoligranulum, Butyricicoccus, Barnesiella*, and *Blautia* in the large intestine. This different spatial distribution of microbiota is likely due to the particular characteristics of each GIT compartment, including the pH, morphological structure, chemical constituents in the gut, the gut motility, and nutrition supply (Holmes et al., 2012; Dolan and Chang, 2017; Espín, 2017; Parker et al., 2018).

In this study, at the KEGG level 2, microbial potential function analysis indicated that the most prominent functional categories were the gene families associated with the functions of membrane transport, carbohydrate metabolism, amino acid metabolism, replication and repair, energy metabolism and translation, which matches the conventional metabolic function (e.g., carbohydrate, protein, and amino acid metabolism) that are indispensable for microbial subsistence (Lamendella et al., 2011). Based on the PCoA results and the comparison of six dominant gene families, we can infer that the microbiota may mainly involve in protein synthesis and metabolism in rumen and duodenum while the majority of bacterial community participate the absorption and utilization of nutrients in hindgut, which is agreement with previous studies on camel and porcine (Zhao et al., 2015; He et al., 2018); however, a report in adult dairy cattle reported the opposite results (Mao et al., 2015). This is likely related to the difference of study object; in this study, the kids underwent a transition process from pre-ruminant to ruminant animals, in which the age also played an import role for the microbiota in each GIT compartment, especially in the rumen. Here, the functional categories of membrane transport and amino acid metabolism were replaced by replication and repair, translation, and energy metabolism as kids aged. In cecum and colon, the genes related to carbohydrate metabolism, energy metabolism, and translation increased while the gene families associated to amino acid metabolism decreased with increasing age. These results reflect that the bacterial community in GIT undergoes a functional conversion as kids aged and the change of available nutrient substrate. And there is a different spatial specificity among each GIT region (mainly in rumen and large intestine) for the functional conversion of microbiota. In addition, the bacterial genus-function interaction heatmap showed that not only several genera participated jointly in one KEGG pathway, but a genus could also take part in multiple KEGG pathways, as shown by the genera *Exiguobacterium, Variovorax*, and *Stenotrophomonas*. The genera *Ruminococcus* and *Bacteroides*, well-known for their characteristic of degrading cellulose or soluble saccharides (Cann et al., 2016; Wang et al., 2019) may also participate in the amino acid metabolism and vitamin synthesis metabolism.

In conclusion, the results of this study suggest that the microbiota and putative metabolic function displayed evident temporal and spatial specificity along the GIT in preweaning goats. The colonization process of the microbiota along different GIT compartments can be divided into three stages: initial, transit, and relative stable phase. Corresponding to the bacterial developmental process, the bacterial genera mainly consist of the leading, transitional, and mature taxa. Each type of bacterial genera differs in each compart. Compared to the bacterial community composition in the rumen, the transit stage of the microbiota in the intestinal tract is slightly longer, where the microbiota did not have access to a relatively stable stage until 42 days of age. Therefore, this suggests that the microbiota in the rumen is more sensitive to the introduction of solid feeds than that of the intestine, which is likely due to the different physiological-biochemical environments of both regions, such as pH, oxygen contents, redox potential, host secretions, and feed particle size. Moreover, the present study supports a more heterogeneous goat microbiota at birth that then inclines to develop into a mature community, which is more homogeneous and specific. This characteristic extends to all GIT regions. Based on these results, probiotic intervention during the weaning transition (6–8 weeks) may be more effective to facilitate permanent microbiota changes and improve kid health, relative to intervention immediately after birth.

## Data Availability

The 16S rRNA data of intestinal samples are available from the National Center for Biotechnology Information (NCBI) under accession No. SRA: SRP195450. And the accession number of ruminal samples in NCBI is SRP119700. The datasets generated for this study can be found in the NCBI Sequence Read Archive accession SRP195450, https://www.ncbi.nlm.nih.gov/sra/?term=SRP195450.

## Ethics Statement

This study was carried out in accordance with the regulations of Instructive Notions with Respect to Caring for Experimental Animals, Ministry of Science and Technology of China. The protocol of animal handling and sampling in this study was approved by the Experimental Animal Management Committee of the Northwest A&F University (No. 2014ZX08008002). All surgeries were performed while animals were anesthetized with thiopental and potassium chloride. All efforts were made to minimize animal suffering.

## Author Contributions

BL, KZ, XW, YY, and YC designed the experiments. BL and KZ performed the experiment. BL, KZ, and CL carried out the microbial data processing and analysis. BL wrote the manuscript. All authors reviewed the manuscript before submission.

### Conflict of Interest Statement

The authors declare that the research was conducted in the absence of any commercial or financial relationships that could be construed as a potential conflict of interest.

## Supplementary Material

Figure S1 Summary of rarefaction results based on operational taxonomic unit (OTUs) (3% divergence) for each sample.

Figure S2 Bacterial composition in samples of rumen, duodenum, jejunum, ileum, cecum, and colon at different ages (at the phylum level).

Figure S3 Bacterial composition along the gastrointestinal tract at different ages (at the genus level).

Figure S4 Alpha diversity of the bacterial community between gastrointestinal tract (GIT) regions in each age group. The richness and diversity were calculated via Chao **(A)** and Shannon **(B)** indexes, respectively. Boxplots indicate significant differences among GIT region in a given age group.

Figure S5 Bacterial composition in different gastrointestinal tract compartments and comparison of predominant taxa. **(A)** Bacterial composition among different GIT compartments in each age group. **(B)** Comparison of relative abundances of the main bacterial phyla (Firmicutes, Bacteroidetes, and Proteobacteria) among different GIT compartments in each age group. Bars with a star symbol above their whiskers are significantly different between age groups in each GIT compartment using a one-way ANOVA analysis; ^*^0.01 < *P* < 0.05; ^**^0.001 < *P* < 0.01; ^***^*P* < 0.001.

Table S1 Ingredients and nutrients of the experimental diets.

Table S2 Number of sequences, operational taxonomic unit (OTU), richness, diversity, and estimated sample coverage of each sample.

Table S3 Alpha diversity of bacterial community according to ages in each gastrointestinal tract (GIT) region. Mean values with different superscripted lowercase letters within the same column differ significantly (P < 0.05). The same as below in Table S6.

Table S4 Analysis of similarity (ANOSIM) of bacterial microbiota according to age in each gastrointestinal tract (GIT) region. This analysis provides a way to statistically test whether there is a significant difference between two or more groups of samples, by generating a similarity value (R-value) between 0 and 1 using the Bray-Curtis index. R-values closer to 0 represent groups that do not significantly differ, while values closer to 1 represent a highly different community composition. All ANOSIM values were significantly different between the groups at P < 0.01. The same as below in Table S7.

Table S5 Significant difference of 23 genera at different ages in rumen, duodenum, jejunum, ileum, cecum, and colon. Mean values with different superscripted lowercase letters within the same row differ significantly (P < 0.05).

Table S6 Alpha diversity of the bacterial community according to different gastrointestinal tract regions at a given age.

Table S7 Analysis of similarity (ANOSIM) of bacterial community according to GIT region in each age group.

Table S8 Significant difference of 23 genera at different gastrointestinal tract (GIT) regions in each age group.

Table S9 The nearest sequenced taxon index (NSTI) for each sample. The NSTI is used to measure the dissimilarity between the predicted metagenome presented here and reference genomes.

Table S10 Comparisons of the six predominant KEGG gene pathways of bacterial microbiota throughout the gastrointestinal tract (GIT) regions as kids aged. Mean values with different superscripted lowercase letters within the same row differed significantly (P < 0.05). SEM = standard error of the mean.

Table S11 36 predominant KEGG orthologs pathways (at KEGG level 3) along the gastrointestinal tract regions at the different ages.
