# Supplementary figures and images for: Characterization and Comparison of Microbiota in the Gastrointestinal Tracts of the Goat (Capra hircus) During Preweaning Development
Source: Front Microbiol. 2019 Sep 13;10:2125. doi: 10.3389/fmicb.2019.02125 (PMC6753876; doi:10.3389/fmicb.2019.02125)

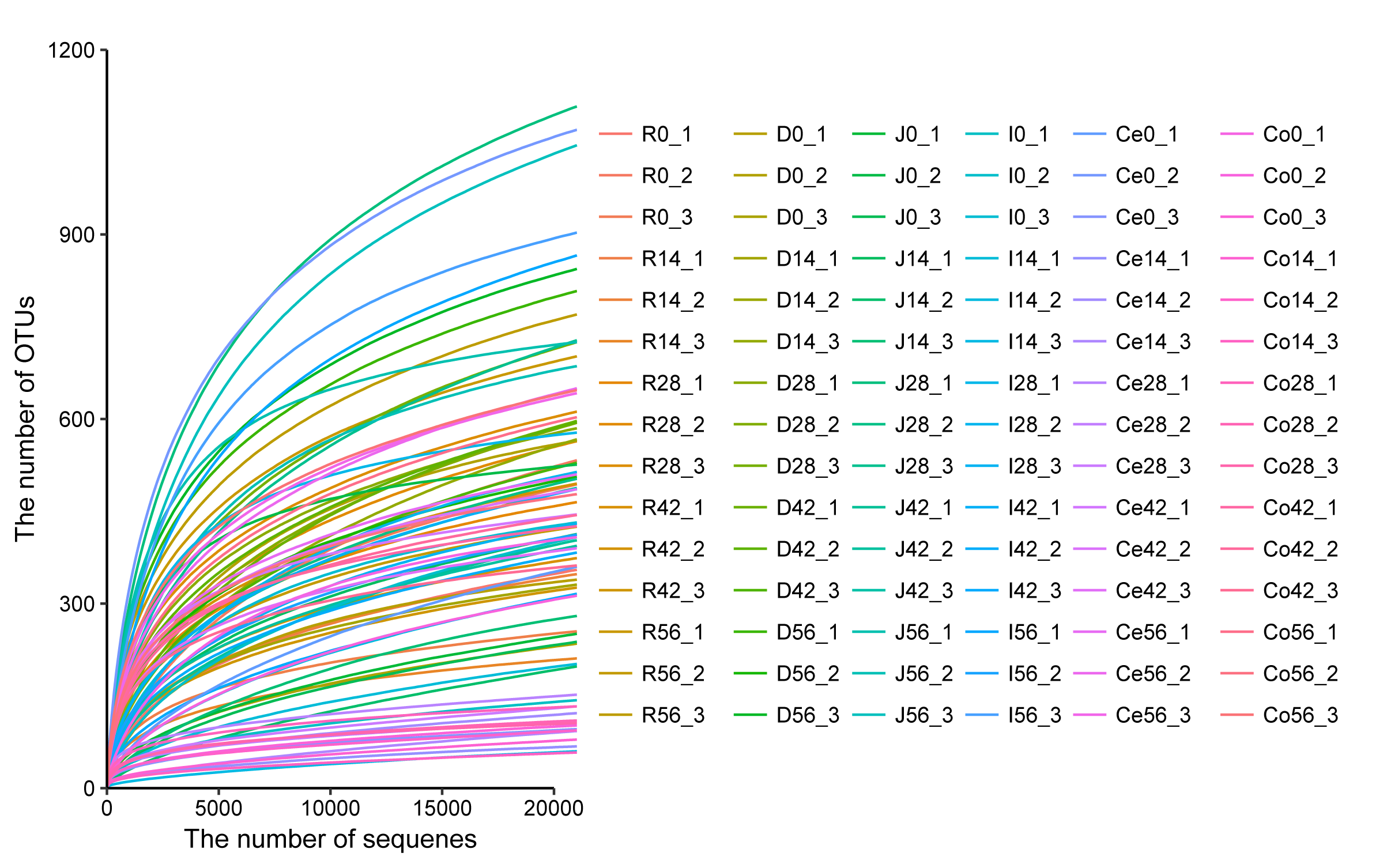

Supplement: Figure S1 — Summary of rarefaction results based on operational taxonomic unit (OTUs) (3% divergence) for each sample. [file Image_1.TIF]

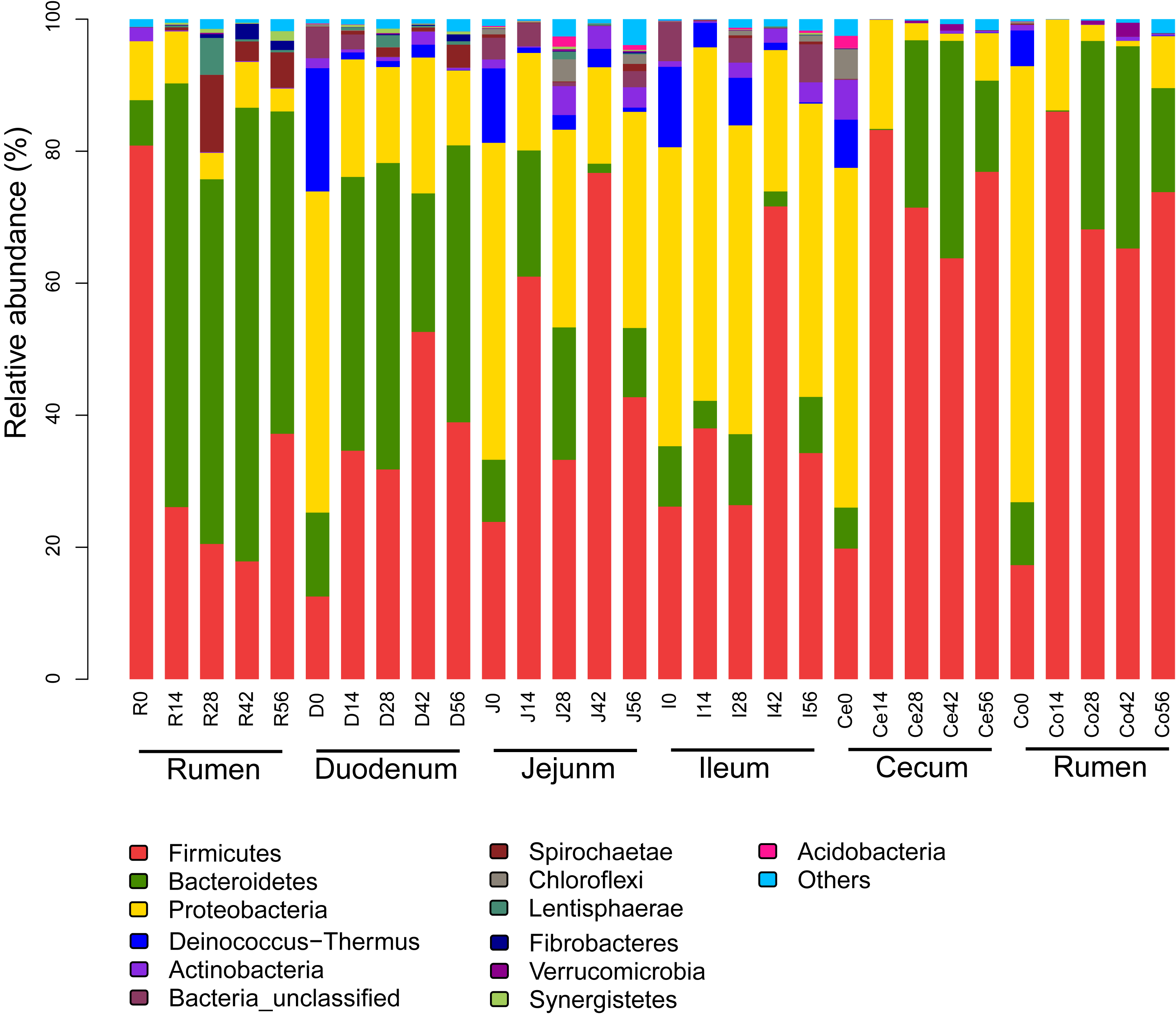

Supplement: Figure S2 — Bacterial composition in samples of rumen, duodenum, jejunum, ileum, cecum, and colon at different ages (at the phylum level). [file Image_2.TIF]

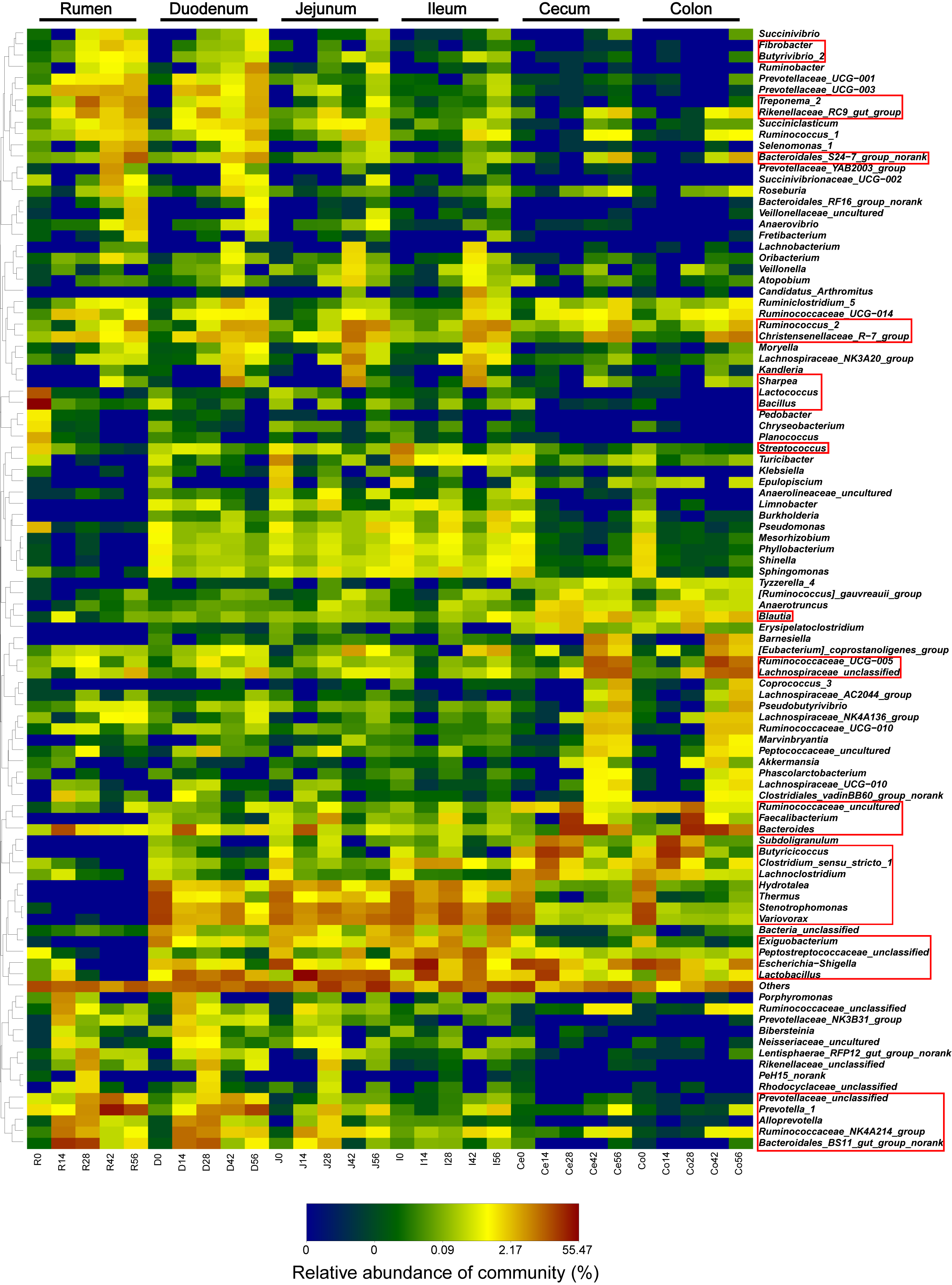

Supplement: Figure S3 — Bacterial composition along the gastrointestinal tract at different ages (at the genus level). [file Image_3.TIF]

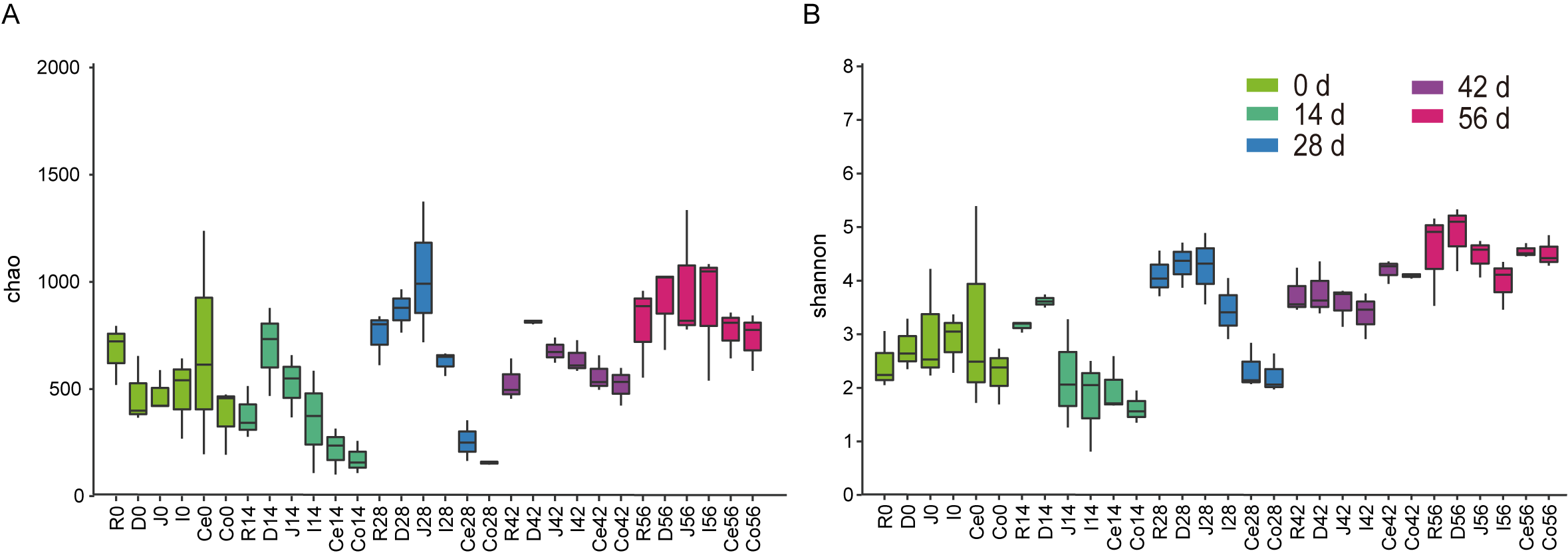

Supplement: Figure S4 — Alpha diversity of the bacterial community between gastrointestinal tract (GIT) regions in each age group. The richness and diversity were calculated via Chao (A) and Shannon (B) indexes, respectively. Boxplots indicate significant differences among GIT region in a given age group. [file Image_4.tif]

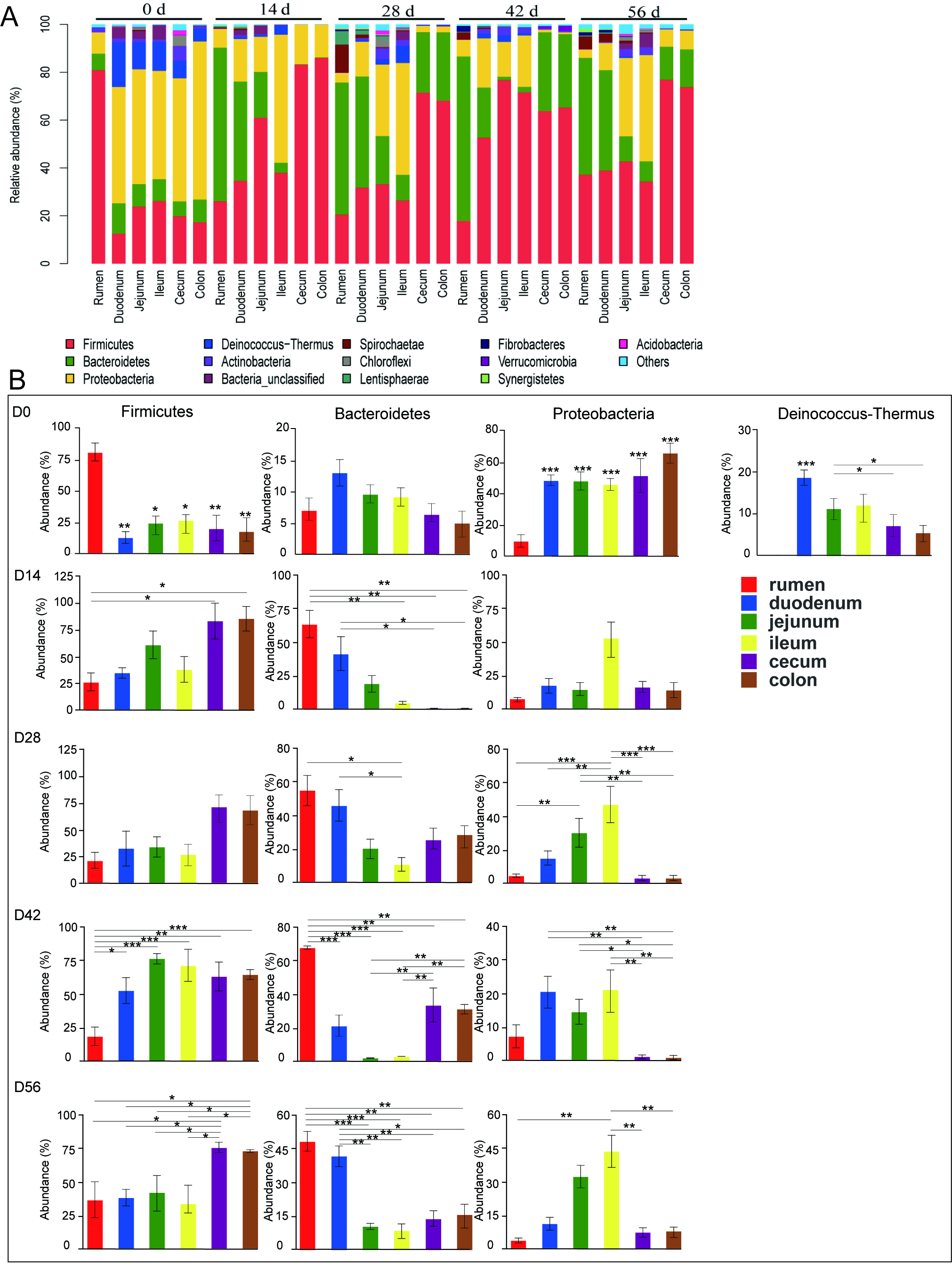

Supplement: Figure S5 — Bacterial composition in different gastrointestinal tract compartments and comparison of predominant taxa. (A) Bacterial composition among different GIT compartments in each age group. (B) Comparison of relative abundances of the main bacterial phyla (Firmicutes, Bacteroidetes, and Proteobacteria) among different GIT compartments in each age group. Bars with a star symbol above their whiskers are significantly different between age groups in each GIT compartment using a one-way ANOVA analysis; *0.01 < P < 0.05; **0.001 < P < 0.01; ***P < 0.001. [file Image_5.TIF]
