# Supplementary material for: Characterization and Comparison of Microbiota in the Gastrointestinal Tracts of the Goat (Capra hircus) During Preweaning Development
Source: Front Microbiol. 2019 Sep 13;10:2125. doi: 10.3389/fmicb.2019.02125 (PMC6753876; doi:10.3389/fmicb.2019.02125)
Supplement: Table S1 — Ingredients and nutrients of the experimental diets. [file Table_1.DOC]

**Table S1 Ingredients and nutrients of the experimental diets.**

| **Ingredients** | **Content (%)** | **Nutrient level** | **Content** |
| --- | --- | --- | --- |
| Corn | 23.30 | Dry matter (DM), % | 89.09 |
| Soybean meal | 13.75 | Digestible energy(MJ/kg） | 8.50 |
| Rice straw | 20.40 | Crude protein, % | 10.00 |
| Straw | 39.60 | crude fat , % | 2.02 |
| Limestone | 0.55 | crude fiber, % | 17.28 |
| Ca(HCO_3_)_2_ | 0.90 | NFE, % | 49.20 |
| NaCl | 0.50 | Ash, % | 8.36 |
| Premix | 1.00 | Calcium, % | 0.70 |
| Total | 100 | Phosphorus, % | 0.40 |

The main nutrients of alfalfa: crude protein 18.1%, lysine 1.76%, methionine + cystine 0.46%, calcium 1.5%, phosphorus 0.49%。
