# Supplementary material for: Characterization and Comparison of Microbiota in the Gastrointestinal Tracts of the Goat (Capra hircus) During Preweaning Development
Source: Front Microbiol. 2019 Sep 13;10:2125. doi: 10.3389/fmicb.2019.02125 (PMC6753876; doi:10.3389/fmicb.2019.02125)
Supplement: Table S2 — Number of sequences, operational taxonomic unit (OTU), richness, diversity, and estimated sample coverage of each sample. [file Table_2.DOCX]

**Table S2 Number of sequences, operational taxonomic unit (OTU), richness, diversity, and estimated sample coverage of each sample.**

| GIT | Age/d | Sample ID | Valid sequences | Subsampling sequences | OTUs | Chao | Shannon | Coverage |
| --- | --- | --- | --- | --- | --- | --- | --- | --- |
| Rumen | 0 | R0_1 | 35804 | 23200 | 355 | 519 | 2.05 | 0.9934 |
|  | 0 | R0_2 | 41006 | 23200 | 533 | 794 | 2.24 | 0.9894 |
|  | 0 | R0_3 | 36195 | 23200 | 494 | 722 | 3.06 | 0.9912 |
|  | 14 | R14_1 | 35327 | 23200 | 255 | 342 | 3.22 | 0.9963 |
|  | 14 | R14_2 | 44841 | 23200 | 348 | 514 | 3.2 | 0.9939 |
|  | 14 | R14_3 | 32324 | 23200 | 211 | 277 | 3.03 | 0.9970 |
|  | 28 | R28_1 | 36592 | 23200 | 465 | 611 | 4.04 | 0.9932 |
|  | 28 | R28_2 | 39959 | 23200 | 564 | 839 | 3.71 | 0.9905 |
|  | 28 | R28_3 | 43261 | 23200 | 612 | 802 | 4.56 | 0.9912 |
|  | 42 | R42_1 | 33138 | 23200 | 374 | 496 | 3.56 | 0.9942 |
|  | 42 | R42_2 | 38301 | 23200 | 495 | 642 | 4.24 | 0.9926 |
|  | 42 | R42_3 | 40788 | 23200 | 326 | 455 | 3.46 | 0.9947 |
|  | 56 | R56_1 | 37329 | 23200 | 702 | 887 | 4.91 | 0.9911 |
|  | 56 | R56_2 | 33776 | 23200 | 425 | 553 | 3.53 | 0.9941 |
|  | 56 | R56_3 | 41015 | 23200 | 770 | 958 | 5.16 | 0.9899 |
| Duodenum | 0 | D0_1 | 41820 | 23200 | 339 | 399 | 2.64 | 0.9958 |
|  | 0 | D0_2 | 33568 | 23200 | 565 | 654 | 3.29 | 0.9933 |
|  | 0 | D0_3 | 32857 | 23200 | 235 | 366 | 2.35 | 0.9950 |
|  | 14 | D14_1 | 40777 | 23200 | 331 | 468 | 3.5 | 0.9948 |
|  | 14 | D14_2 | 30962 | 23200 | 488 | 733 | 3.74 | 0.9913 |
|  | 14 | D14_3 | 40713 | 23200 | 567 | 878 | 3.61 | 0.9882 |
|  | 28 | D28_1 | 36560 | 23200 | 585 | 763 | 4.37 | 0.9917 |
|  | 28 | D28_2 | 37307 | 23200 | 594 | 879 | 3.87 | 0.9890 |
|  | 28 | D28_3 | 38858 | 23200 | 725 | 965 | 4.71 | 0.9887 |
|  | 42 | D42_1 | 33492 | 23200 | 597 | 818 | 3.39 | 0.9892 |
|  | 42 | D42_2 | 39290 | 23200 | 595 | 819 | 4.36 | 0.9900 |
|  | 42 | D42_3 | 37931 | 23200 | 529 | 802 | 3.63 | 0.9901 |
|  | 56 | D56_1 | 39140 | 23200 | 808 | 1021 | 5.1 | 0.9896 |
|  | 56 | D56_2 | 42908 | 23200 | 506 | 683 | 4.18 | 0.9924 |
|  | 56 | D56_3 | 41019 | 23200 | 844 | 1027 | 5.33 | 0.9897 |
| Jejunum | 0 | J0_1 | 31535 | 23200 | 251 | 422 | 2.53 | 0.9941 |
|  | 0 | J0_2 | 37352 | 23200 | 526 | 588 | 4.22 | 0.9964 |
|  | 0 | J0_3 | 36040 | 23200 | 238 | 419 | 2.23 | 0.9943 |
|  | 14 | J14_1 | 38033 | 23200 | 413 | 658 | 3.28 | 0.9922 |
|  | 14 | J14_2 | 31109 | 23200 | 198 | 549 | 1.26 | 0.9938 |
|  | 14 | J14_3 | 42032 | 23200 | 280 | 368 | 2.06 | 0.9942 |
|  | 28 | J28_1 | 44571 | 23200 | 1108 | 1374 | 4.89 | 0.9857 |
|  | 28 | J28_2 | 39936 | 23200 | 503 | 718 | 3.56 | 0.9910 |
|  | 28 | J28_3 | 33323 | 23200 | 728 | 991 | 4.32 | 0.9879 |
|  | 42 | J42_1 | 36113 | 23200 | 403 | 623 | 3.14 | 0.9916 |
|  | 42 | J42_2 | 31565 | 23200 | 394 | 673 | 3.75 | 0.9924 |
|  | 42 | J42_3 | 36636 | 23200 | 488 | 740 | 3.81 | 0.9913 |
|  | 56 | J56_1 | 34133 | 23200 | 725 | 778 | 4.74 | 0.9957 |
|  | 56 | J56_2 | 38260 | 23200 | 686 | 818 | 4.06 | 0.9923 |
|  | 56 | J56_3 | 38689 | 23200 | 1045 | 1335 | 4.58 | 0.9861 |
| Ileum | 0 | I0_1 | 32336 | 23200 | 143 | 269 | 2.28 | 0.9970 |
|  | 0 | I0_2 | 39608 | 23200 | 408 | 642 | 3.05 | 0.9914 |
|  | 0 | I0_3 | 41459 | 23200 | 432 | 541 | 3.37 | 0.9944 |
|  | 14 | I14_1 | 36112 | 23200 | 202 | 374 | 2.05 | 0.9951 |
|  | 14 | I14_2 | 40635 | 23200 | 316 | 585 | 2.5 | 0.9925 |
|  | 14 | I14_3 | 40386 | 23200 | 60 | 108 | 0.81 | 0.9983 |
|  | 28 | I28_1 | 39388 | 23200 | 578 | 650 | 4.05 | 0.9957 |
|  | 28 | I28_2 | 32980 | 23200 | 514 | 666 | 2.91 | 0.9918 |
|  | 28 | I28_3 | 43411 | 23200 | 430 | 561 | 3.41 | 0.9931 |
|  | 42 | I42_1 | 32368 | 23200 | 486 | 728 | 3.76 | 0.9915 |
|  | 42 | I42_2 | 31698 | 23200 | 383 | 585 | 3.46 | 0.9927 |
|  | 42 | I42_3 | 31569 | 23200 | 316 | 610 | 2.91 | 0.9925 |
|  | 56 | I56_1 | 34353 | 23200 | 866 | 1083 | 4.11 | 0.9886 |
|  | 56 | I56_2 | 35533 | 23200 | 412 | 539 | 3.46 | 0.9934 |
|  | 56 | I56_3 | 39402 | 23200 | 903 | 1049 | 4.35 | 0.9908 |
| Cecum | 0 | Ce0_1 | 39166 | 23200 | 360 | 614 | 2.49 | 0.9913 |
|  | 0 | Ce0_2 | 42775 | 23200 | 1070 | 1238 | 5.39 | 0.9882 |
|  | 0 | Ce0_3 | 33135 | 23200 | 96 | 196 | 1.72 | 0.9981 |
|  | 14 | Ce14_1 | 43960 | 23200 | 68 | 101 | 1.67 | 0.9985 |
|  | 14 | Ce14_2 | 35952 | 23200 | 122 | 315 | 2.59 | 0.9972 |
|  | 14 | Ce14_3 | 42947 | 23200 | 93 | 236 | 1.71 | 0.9973 |
|  | 28 | Ce28_1 | 36223 | 23200 | 152 | 250 | 2.84 | 0.9974 |
|  | 28 | Ce28_2 | 40446 | 23200 | 109 | 165 | 2.14 | 0.9981 |
|  | 28 | Ce28_3 | 40134 | 23200 | 133 | 354 | 2.07 | 0.9970 |
|  | 42 | Ce42_1 | 32975 | 23200 | 445 | 532 | 4.36 | 0.9956 |
|  | 42 | Ce42_2 | 41399 | 23200 | 486 | 657 | 4.27 | 0.9934 |
|  | 42 | Ce42_3 | 42178 | 23200 | 390 | 497 | 3.94 | 0.9953 |
|  | 56 | Ce56_1 | 44823 | 23200 | 510 | 643 | 4.51 | 0.9931 |
|  | 56 | Ce56_2 | 38621 | 23200 | 650 | 856 | 4.45 | 0.9903 |
|  | 56 | Ce56_3 | 38130 | 23200 | 642 | 809 | 4.7 | 0.9916 |
| Colon | 0 | Co0_1 | 34515 | 23200 | 408 | 474 | 2.73 | 0.9953 |
|  | 0 | Co0_2 | 37610 | 23200 | 313 | 457 | 2.38 | 0.9933 |
|  | 0 | Co0_3 | 30835 | 23200 | 102 | 193 | 1.69 | 0.9979 |
|  | 14 | Co14_1 | 37066 | 23200 | 79 | 157 | 1.95 | 0.9980 |
|  | 14 | Co14_2 | 30242 | 23200 | 94 | 258 | 1.56 | 0.9979 |
|  | 14 | Co14_3 | 32694 | 23200 | 58 | 108 | 1.35 | 0.9986 |
|  | 28 | Co28_1 | 31183 | 23200 | 133 | 164 | 2.64 | 0.9983 |
|  | 28 | Co28_2 | 37380 | 23200 | 110 | 157 | 2.06 | 0.9983 |
|  | 28 | Co28_3 | 32245 | 23200 | 106 | 147 | 1.97 | 0.9985 |
|  | 42 | Co42_1 | 31393 | 23200 | 426 | 533 | 4.1 | 0.9954 |
|  | 42 | Co42_2 | 39285 | 23200 | 444 | 598 | 4.14 | 0.9941 |
|  | 42 | Co42_3 | 42889 | 23200 | 362 | 423 | 4.04 | 0.9963 |
|  | 56 | Co56_1 | 31039 | 23200 | 603 | 776 | 4.28 | 0.9913 |
|  | 56 | Co56_2 | 44581 | 23200 | 478 | 585 | 4.42 | 0.9942 |
|  | 56 | Co56_3 | 43039 | 23200 | 647 | 843 | 4.85 | 0.9914 |
