# Supplementary material for: Characterization and Comparison of Microbiota in the Gastrointestinal Tracts of the Goat (Capra hircus) During Preweaning Development
Source: Front Microbiol. 2019 Sep 13;10:2125. doi: 10.3389/fmicb.2019.02125 (PMC6753876; doi:10.3389/fmicb.2019.02125)
Supplement: Table S3 — Alpha diversity of bacterial community according to ages in each gastrointestinal tract (GIT) region. Mean values with different superscripted lowercase letters within the same column differ significantly (P < 0.05). The same as below in Table S6. [file Table_3.docx]

**Table S3 Alpha diversity of bacterial community according to ages in each gastrointestinal tract (GIT) region.** Mean values with different superscripted lowercase letters within the same column differ significantly *(P* < 0.05). The same as below in Table S6. ^1^SEM = standard error of mean.

| **Regions** | **Day ages** | **Chao** | **Ace** | **Shannon** | **Simpson** |
| --- | --- | --- | --- | --- | --- |
| Rumen | 0d | 678.33±82.33^b^ | 764.67±87.11 | 2.45±0.31^a^ | 0.33±0.05^b^ |
|  | 14d | 377.67±70.70^a^ | 403.67±98.55 | 3.15±0.06^ab^ | 0.10±0.01^a^ |
|  | 28d | 750.67±70.65^b^ | 724.67±56.01 | 4.10±0.25^bc^ | 0.05±0.02^a^ |
|  | 42d | 531.00±56.75^ab^ | 569.33±91.57 | 3.75±0.24^bc^ | 0.06±0.01^a^ |
|  | 56d | 799.33±124.86^b^ | 787.00±129.40 | 4.53±0.51^c^ | 0.04±0.02^a^ |
|  | *P* | 0.030 | 0.080 | 0.006 | 0.000 |
| Duodenum | 0d | 473.00±91.00^a^ | 509.67±78.22 | 2.76±0.28^a^ | 0.13±0.01^c^ |
|  | 14d | 693.00±120.04^ab^ | 802.33±162.19 | 3.62±0.07^b^ | 0.07±0.00^b^ |
|  | 28d | 869.00±58.53^b^ | 848.67±59.84 | 4.32±0.24^bc^ | 0.04±0.01^ab^ |
|  | 42d | 813.00±5.51^b^ | 810.33±23.67 | 3.79±0.29^b^ | 0.07±0.02^b^ |
|  | 56d | 910.33±113.68^b^ | 900.00±113.21 | 4.87±0.35^c^ | 0.02±0.01^a^ |
|  | *P* | 0.035 | 0.123 | 0.002 | 0.002 |
| Jejunum | 0d | 476.33±55.84^a^ | 556.00±13.45 | 2.99±0.62^ab^ | 0.13±0.05 |
|  | 14d | 525.00±84.57^a^ | 643.00±123.28 | 2.20±0.58^a^ | 0.29±0.12 |
|  | 28d | 1027.67±190.26^b^ | 1050.33±152.71 | 4.26±0.38^b^ | 0.06±0.02 |
|  | 42d | 678.67±22.154^ab^ | 794.67±22.15 | 3.57±0.21^ab^ | 0.07±0.02 |
|  | 56d | 977.00±179.37^b^ | 937.67±161.24 | 4.46±0.21^b^ | 0.06±0.01 |
|  | *P* | 0.033 | 0.063 | 0.025 | 0.069 |
| Ileum | 0d | 484.00±50.38 | 529.00±26.37 | 2.90±0.32^b^ | 0.11±0.03^a^ |
|  | 14d | 355.67±39.00 | 424.67±61.90 | 1.79±0.51^a^ | 0.33±0.12^b^ |
|  | 28d | 625.67±32.66 | 616.33±26.03 | 3.46±0.33^b^ | 0.09±0.02^a^ |
|  | 42d | 641.00±44.10 | 726.33±31.80 | 3.38±0.25^b^ | 0.08±0.02^a^ |
|  | 56d | 890.33±75.94 | 876.00 | 3.97±0.26^b^ | 0.08±0.01^a^ |
|  | *P* | 0.069 | 0.141 | 0.012 | 0.036 |
| Cecum | 0d | 682.67±52.75 | 737.00±69.56 | 3.20±0.55^ab^ | 0.17±0.09 |
|  | 14d | 217.33±62.48 | 295.00±71.68 | 1.99±0.30^a^ | 0.21±0.04 |
|  | 28d | 256.33±54.65 | 352.00±75.03 | 2.35±0.25^a^ | 0.22±0.05 |
|  | 42d | 562.00±48.56 | 535.67±40.75 | 4.19±0.13^b^ | 0.04±0.01 |
|  | 56d | 769.33±64.61 | 765.33±59.49 | 4.55±0.08^b^ | 0.03±0.01 |
|  | *P* | 0.075 | 0.160 | 0.026 | 0.063 |
| Colon | 0d | 374.67±90.97^b^ | 387.67±86.46^bc^ | 2.27±0.311^a^ | 0.23±0.06^b^ |
|  | 14d | 174.33±44.16^a^ | 269.00±45.24^ab^ | 1.62±0.18^a^ | 0.33±0.08^b^ |
|  | 28d | 156.00±4.93^a^ | 176.33±7.84^a^ | 2.22±0.21^a^ | 0.26±0.06^b^ |
|  | 42d | 518.00±51.07^b^ | 497.00±38.80^c^ | 4.09±0.03^b^ | 0.05±0.00^a^ |
|  | 56d | 734.67±77.29^c^ | 724.00±71.31^d^ | 4.52±0.17^b^ | 0.03±0.01^a^ |
|  | *P* | 0.000 | 0.000 | 0.000 | 0.009 |
