# Supplementary material for: Characterization and Comparison of Microbiota in the Gastrointestinal Tracts of the Goat (Capra hircus) During Preweaning Development
Source: Front Microbiol. 2019 Sep 13;10:2125. doi: 10.3389/fmicb.2019.02125 (PMC6753876; doi:10.3389/fmicb.2019.02125)
Supplement: Table S4 — Analysis of similarity (ANOSIM) of bacterial microbiota according to age in each gastrointestinal tract (GIT) region. This analysis provides a way to statistically test whether there is a significant difference between two or more groups of samples, by generating a similarity value (R-value) between 0 and 1 using the Bray-Curtis index. R-values closer to 0 represent groups that do not significantly differ, while values closer to 1 represent a highly different community composition. All ANOSIM values were significantly different between the groups at P < 0.01. The same as below in Table S7. [file Table_4.DOCX]

**Table S4 Analysis of similarity (ANOSIM)** **of bacterial microbiota according to age in each gastrointestinal tract (GIT) region.** This analysis provides a way to statistically test whether there is a significant difference between two or more groups of samples, by generating a similarity value (R-value) between 0 and 1 using the Bray-Curtis index. R-values closer to 0 represent groups that do not significantly differ, while values closer to 1 represent a highly different community composition. All ANOSIM values were significantly different between the groups at *P* < 0.01. The same as below in Table S7.

|  | 0 d | 14 d | 28 d | 42 d | 56 d |  |
| --- | --- | --- | --- | --- | --- | --- |
| **Digestia sample** |  |  |  |  |  |  |
| **Rumen** |  |  |  |  |  | **Duodenum** |
| 0 d |  | 0.963 | 1 | 0.6667 | 1 | 0 d |
| 14 d | 1 |  | 0.4815 | 0.7037 | 1 | 14 d |
| 28 d | 1 | 0.7037 |  | 0.8148 | 0.9259 | 28 d |
| 42d | 1 | 1 | 0.963 |  | 0.6296 | 42d |
| 56 d | 1 | 1 | 0.7778 | 0.2593 |  | 56 d |
| **Jejunum** |  |  |  |  |  | **Ileum** |
| 0 d |  | 0.4444 | 0 | 0.8889 | 0.4815 | 0 d |
| 14 d | 0.6296 |  | 0.5556 | 0.2593 | 0.7778 | 14 d |
| 28 d | 0.3704 | -0.0741 |  | 0.8519 | 0 | 28 d |
| 42d | 0.7778 | 0.5556 | 0.6296 |  | 0.8889 | 42d |
| 56 d | 0.2963 | 0.2222 | -0.037 | 0.3704 |  | 56 d |
| **Cecum** |  |  |  |  |  | **Colon** |
| 0 d |  | 0.5926 | 0.7778 | 1 | 0.9259 | 0 d |
| 14 d | 0.2222 |  | 0.2593 | 1 | 0.8519 | 14 d |
| 28 d | 0.4444 | 0.6296 |  | 0.8519 | 1 | 28 d |
| 42d | 0.4815 | 1 | 0.963 |  | 0.2963 | 42d |
| 56 d | 0.4074 | 0.9259 | 1 | 0.3704 |  | 56 d |
| 42d | 1 | 1 | 0.963 |  |  |  |
| 56 d | 1 | 1 | 1 | 0.1111 |  |  |
