# Supplementary material for: Characterization and Comparison of Microbiota in the Gastrointestinal Tracts of the Goat (Capra hircus) During Preweaning Development
Source: Front Microbiol. 2019 Sep 13;10:2125. doi: 10.3389/fmicb.2019.02125 (PMC6753876; doi:10.3389/fmicb.2019.02125)
Supplement: Table S6 — Alpha diversity of the bacterial community according to different gastrointestinal tract regions at a given age. [file Table_6.docx]

**Table S6 Alpha diversity of the bacterial community according to different gastrointestinal tract regions at a given age.**

| **Day ages** | **Regions** | **Chao** | **Ace** | **Shannon** | **Simpson** |
| --- | --- | --- | --- | --- | --- |
| 0d | Rumen | 678.33±82.33 | 764.67±87.11 | 2.45±0.31 | 0.33±0.05 |
|  | Duodenum | 473.00±91.00 | 509.67±78.22 | 2.76±0.28 | 0.13±0.01 |
|  | Jejunum | 476.33±55.84 | 556.00±13.45 | 2.99±0.62 | 0.13±0.05 |
|  | Ileum | 484.00±55.38 | 529.00±63.37 | 2.90±0.32 | 0.11±0.03 |
|  | Cecum | 682.67±62.75 | 737.00±61.56 | 3.20±0.56 | 0.17±0.09 |
|  | Colon | 374.67±90.97 | 387.67±86.46 | 2.27±0.31 | 0.23±0.07 |
|  | *P* | 0.625 | 0.492 | 0.864 | 0.134 |
| 14d | Rumen | 377.67±70.70^ab^ | 403.67±98.55 | 3.15±0.06^bc^ | 0.10±0.01 |
|  | Duodenum | 693.00±60.04^c^ | 802.33±81.18 | 3.62±0.07^c^ | 0.07±0.00 |
|  | Jejunum | 525.00±84.57^bc^ | 643.00±61.28 | 2.20±0.59^ab^ | 0.29±0.12 |
|  | Ileum | 355.67±69.00^ab^ | 424.67±80.89 | 1.79±0.51^a^ | 0.33±0.12 |
|  | Cecum | 217.33±62.48^a^ | 295.00±71.68 | 1.99±0.30^a^ | 0.21±0.04 |
|  | Colon | 174.33±44.16^a^ | 269.00±45.24 | 1.62±0.18^a^ | 0.33±0.08 |
|  | *P* | 0.017 | 0.051 | 0.008 | 0.117 |
| 28d | Rumen | 750.67±70.65^bc^ | 724.67±56.01^b^ | 4.10±0.25^b^ | 0.05±0.02^a^ |
|  | Duodenum | 869.00±58.53^bc^ | 848.67±59.84^bc^ | 4.32±0.24^b^ | 0.04±0.01^a^ |
|  | Jejunum | 1027.67±95.26^c^ | 1050.33±76.12^c^ | 4.26±0.39^b^ | 0.06±0.01^a^ |
|  | Ileum | 625.67±32.66^b^ | 616.33±26.03^b^ | 3.46±0.33^b^ | 0.09±0.02^a^ |
|  | Cecum | 256.33±54.65^a^ | 352.00±75.02^a^ | 2.35±0.25^a^ | 0.22±0.05^b^ |
|  | Colon | 156.00±4.93^a^ | 176.33±7.84^a^ | 2.22±0.21^a^ | 0.26±0.06^b^ |
|  | *P* | 0.000 | 0.000 | 0.000 | 0.002 |
| 42d | Rumen | 531.00±56.75^a^ | 569.33±91.57^a^ | 3.75±0.25 | 0.06±0.01 |
|  | Duodenum | 813.00±23.67^c^ | 810.33±23.67^b^ | 3.79±0.29 | 0.07±0.02 |
|  | Jejunum | 678.67±33.89^b^ | 794.67±22.15^b^ | 3.57±0.21 | 0.07±0.02 |
|  | Ileum | 641.00±44.10^ab^ | 726.33±31.80^b^ | 3.38±0.25 | 0.08±0.02 |
|  | Cecum | 562.00±48.86^ab^ | 535.67±40.75^a^ | 4.19±0.13 | 0.04±0.01 |
|  | Colon | 518.00±51.07^a^ | 497.00±38.80^a^ | 4.09±0.03 | 0.05±0.00 |
|  | *P* | 0.003 | 0.001 | 0.135 | 0.504 |
| 56d | Rumen | 799.33±62.43 | 787.00±64.20 | 4.53±0.51 | 0.04±0.02 |
|  | Duodenum | 910.33±56.84 | 900.00±56.61 | 4.87±0.35 | 0.02±0.01 |
|  | Jejunum | 977.00±89.69 | 937.67±80.62 | 4.46±0.21 | 0.06±0.01 |
|  | Ileum | 890.33±87.47 | 876.00±79.58 | 3.97±0.27 | 0.08±0.01 |
|  | Cecum | 769.33±64.61 | 765.33±59.49 | 4.55±0.08 | 0.03±0.01 |
|  | Colon | 734.67±77.29 | 724.00±71.31 | 4.52±0.17 | 0.03±0.01 |
|  | *P* | 0.760 | 0.784 | 0.484 | 0.063 |
