# Supplementary material for: Characterization and Comparison of Microbiota in the Gastrointestinal Tracts of the Goat (Capra hircus) During Preweaning Development
Source: Front Microbiol. 2019 Sep 13;10:2125. doi: 10.3389/fmicb.2019.02125 (PMC6753876; doi:10.3389/fmicb.2019.02125)
Supplement: Table S7 — Analysis of similarity (ANOSIM) of bacterial community according to GIT region in each age group. [file Table_7.DOCX]

**Table S7 Analysis of similarity (ANOSIM) of bacterial community according to GIT region in each age group.**

|  | Rumen | Duodenum | Jejunum | Ileum | Cecum | Colon |  |
| --- | --- | --- | --- | --- | --- | --- | --- |
| **0 d** |  |  |  |  |  |  | **14 d** |
| Rumen |  | -0.0741 | 0.6667 | 1 | 1 | 1 | Rumen |
| Duodenum | 1 |  | -0.0741 | 0.5556 | 0.8148 | 0.7407 | Duodenum |
| Jejunum | 0.105 | 0.1481 |  | 0.2593 | 0.4815 | 0.5926 | Jejunum |
| Ileum | 1 | 0.037 | -0.2963 |  | 0.2963 | 0.2963 | Ileum |
| Cecum | 0.5926 | 0.0741 | -0.2593 | 0 |  | -0.2593 | Cecum |
| Colon | 1 | 0.4441 | -0.1481 | 0.037 | -0.2222 |  | Colon |
| **28 d** |  |  |  |  |  |  | **42 d** |
| Rumen |  | 0.6667 | 1 | 1 | 1 | 1 | Rumen |
| Duodenum | 0 |  | -0.1111 | 0.1852 | 1 | 1 | Duodenum |
| Jejunum | 0.5185 | -0.1852 |  | -0.4074 | 1 | 1 | Jejunum |
| Ileum | 1 | 0.963 | 0.2963 |  | 1 | 1 | Ileum |
| Cecum | 1 | 1 | 0.963 | 1 |  | -0.3704 | Cecum |
| Colon | 1 | 1 | 0.8889 | 1 | -0.2593 |  | Colon |
| **56 d** |  |  |  |  |  |  |  |
| Rumen |  |  |  |  |  |  |  |
| Duodenum | -0.481 |  |  |  |  |  |  |
| Jejunum | 0.6667 | 0.4444 |  |  |  |  |  |
| Ileum | 0.8519 | 0.8889 | 0.0741 |  |  |  |  |
| Cecum | 1 | 1 | 1 | 1 |  |  |  |
| Colon | 1 | 1 | 1 | 1 | -0.3704 |  |  |
