# Supplementary material for: Characterization and Comparison of Microbiota in the Gastrointestinal Tracts of the Goat (Capra hircus) During Preweaning Development
Source: Front Microbiol. 2019 Sep 13;10:2125. doi: 10.3389/fmicb.2019.02125 (PMC6753876; doi:10.3389/fmicb.2019.02125)
Supplement: Table S10 — Comparisons of the six predominant KEGG gene pathways of bacterial microbiota throughout the gastrointestinal tract (GIT) regions as kids aged. Mean values with different superscripted lowercase letters within the same row differed significantly (P < 0.05). SEM = standard error of the mean. [file Table_10.DOCX]

**Table S10 Comparisons of the six predominant KEGG gene pathways of bacterial microbiota throughout the gastrointestinal tract (GIT) regions as kids aged.** Mean values with different superscripted lowercase letters within the same row differed significantly *(P* < 0.05). SEM = standard error of the mean.

| **Category** | **Ages group** | | | | | **SEM** | ***P*** |
| --- | --- | --- | --- | --- | --- | --- | --- |
|  | **0 d** | **14 d** | **28 d** | **42 d** | **56 d** |  |  |
| **Rumen** |  |  |  |  |  |  |  |
| Carbohydrate Metabolism | 10.93 | 10.28 | 9.81 | 10.23 | 10.49 | 0.14 | 0.140 |
| Amino Acid Metabolism | 10.03 | 10.12 | 10.08 | 10.46 | 10.32 | 0.10 | 0.666 |
| Membrane Transport | 12.71^b^ | 8.44^a^ | 9.70^a^ | 8.91^a^ | 10.08^a^ | 0.48 | 0.015 |
| Replication and Repair | 8.30^a^ | 9.53^b^ | 9.70^b^ | 9.76^b^ | 9.82^b^ | 0.18 | 0.008 |
| Translation | 5.34^a^ | 6.12^b^ | 6.32^b^ | 6.24^b^ | 6.30^b^ | 0.12 | 0.026 |
| Energy Metabolism | 5.04^b^ | 6.06^b^ | 5.95^b^ | 5.94^b^ | 5.64^b^ | 0.11 | 0.002 |
| **Duodenum** |  |  |  |  |  |  |  |
| Membrane Transport | 11.36^b^ | 9.54^a^ | 10.01^a^ | 11.48^b^ | 10.25^a^ | 0.23 | 0.003 |
| Carbohydrate Metabolism | 10.33 | 10.27 | 9.93 | 10.75 | 10.33 | 0.10 | 0.083 |
| Amino Acid Metabolism | 11.17^b^ | 9.75^a^ | 9.92^a^ | 9.85^a^ | 10.17^a^ | 0.17 | 0.012 |
| Replication and Repair | 7.46^a^ | 9.19^b^ | 9.2^b^ | 9.11^b^ | 9.71^b^ | 0.22 | 0.001 |
| Energy Metabolism | 5.76 | 5.83 | 5.85 | 5.46 | 5.71 | 0.05 | 0.117 |
| Translation | 4.62^a^ | 5.84^b^ | 5.92^b^ | 5.82^b^ | 6.23^b^ | 0.16 | 0.001 |
| **Jejunum** |  |  |  |  |  |  |  |
| Membrane Transport | 12.92 | 11.25 | 10.81 | 12.67 | 11.01 | 0.33 | 0.088 |
| Carbohydrate Metabolism | 10.18 | 10.64 | 10.30 | 10.83 | 10.54 | 0.12 | 0.471 |
| Amino Acid Metabolism | 10.00 | 8.30 | 10.22 | 9.76 | 10.10 | 0.26 | 0.102 |
| Replication and Repair | 7.65 | 9.85 | 8.20 | 9.20 | 8.53 | 0.28 | 0.095 |
| Energy Metabolism | 5.54 | 5.51 | 5.69 | 5.34 | 5.54 | 0.07 | 0.689 |
| Translation | 4.67 | 6.20 | 5.10 | 5.96 | 5.37 | 0.2 | 0.055 |
| **Ileum** |  |  |  |  |  |  |  |
| Membrane Transport | 12.27 | 12.66 | 11.41 | 12.56 | 11.43 | 0.25 | 0.072 |
| Carbohydrate Metabolism | 10.12 | 9.47 | 10.07 | 10.31 | 10.35 | 0.16 | 0.104 |
| Amino Acid Metabolism | 10.45 | 8.78 | 10.54 | 9.52 | 10.16 | 0.25 | 0.156 |
| Replication and Repair | 7.69^a^ | 8.68^ab^ | 7.69^a^ | 9.11^b^ | 8.21^a^ | 0.23 | 0.022 |
| Energy Metabolism | 5.52 | 5.40 | 5.63 | 5.32 | 5.45 | 0.07 | 0.857 |
| Translation | 4.67^a^ | 5.24^ab^ | 4.71^a^ | 5.87^b^ | 5.06^a^ | 0.17 | 0.009 |
| **Cecum** |  |  |  |  |  |  |  |
| Membrane Transport | 12.86 | 14.68 | 12.58 | 12.58 | 13.38 | 0.33 | 0.235 |
| Carbohydrate Metabolism | 10.31 | 10.86 | 11.08 | 10.88 | 10.39 | 0.18 | 0.660 |
| Amino Acid Metabolism | 10.21 | 8.79 | 9.72 | 9.49 | 9.57 | 0.18 | 0.171 |
| Replication and Repair | 7.68 | 8.83 | 8.87 | 8.59 | 8.76 | 0.18 | 0.173 |
| Energy Metabolism | 5.61 | 5.60 | 5.84 | 5.82 | 5.64 | 0.05 | 0.234 |
| Translation | 4.58^a^ | 5.48^b^ | 5.59^b^ | 5.41^b^ | 5.64^b^ | 0.13 | 0.018 |
| **Colon** |  |  |  |  |  |  |  |
| Membrane Transport | 12.45 | 14.66 | 12.59 | 12.50 | 13.33 | 0.31 | 0.094 |
| Carbohydrate Metabolism | 10.03 | 10.71 | 11.24 | 10.71 | 10.36 | 0.16 | 0.172 |
| Amino Acid Metabolism | 10.07 | 8.94 | 9.68 | 9.46 | 9.55 | 0.15 | 0.216 |
| Replication and Repair | 7.78 | 8.91 | 8.81 | 8.59 | 8.75 | 0.15 | 0.085 |
| Energy Metabolism | 5.6^ab^ | 5.49^a^ | 5.84^c^ | 5.82^bc^ | 5.64^abc^ | 0.04 | 0.021 |
| Translation | 4.52^a^ | 5.56^b^ | 5.52^b^ | 5.43^b^ | 5.62^b^ | 0.13 | 0.006 |
